# Screening for Autism Spectrum Disorder in Young Children: Still Not Enough Evidence

**DOI:** 10.1177/21501319241263223

**Published:** 2024-07-30

**Authors:** Bogdan Grigore, Jaime Peters, Jessica Williams, Ginny Russell, Paula Coles, Cristina Visintin, Morwenna Rogers, Robert Hayward, Zhivko Zhelev, Stuart Logan, Christopher Hyde

**Affiliations:** 1 University of Exeter, Exeter, UK; 2 UK National Screening Committee, London, UK; 3 South Gloucestershire Council, Bristol, UK

**Keywords:** autism spectrum disorder, ASD, screening, early detection, early intervention

## Abstract

**Background:**

Early detection of autism spectrum disorder (ASD) has the potential to significantly reduce the impact of the condition, however previous reviews have found little evidence to support screening programs for ASD in young children.

**Methods:**

We conducted a review with the aim of updating evidence on 3 aspects: (a) diagnostic stability of ASD in young children; (b) accuracy of ASD screening tools in young children; and (c) the benefits of early interventions in screen-detected young children with ASD.

**Results:**

A total of 33 studies were included in our review. Five studies looking at diagnostic stability reported estimates ranging from 71.9% to 100%, however the majority only included a follow-up of 24 months and all studies raised concerns regarding the risk of bias due particularly to lack of blinding, sample size, and patient flow. A total of 25 studies, reported in 26 articles, were identified that reported accuracy data on 11 screening tools. Most of the reports were concerned with versions of M-CHAT, reporting sensitivity estimates from 0.67 to 1.0; however, many of these were deemed to be of high risk of bias due to lack of blinding and follow-up. Four studies reported on early interventions in screen-detected children; however, the majority did not find significant improvements on the relevant outcomes.

**Conclusions:**

Overall, the evidence on screening for ASD in young children captured by this review is not conclusive regarding the 3 aspects of screening in this population. Future studies should attempt to ensure blinded diagnostic assessments, include longer follow-up periods and limit attrition.

## Background

Autism spectrum disorder (ASD) describes a range of of neurodevelopmental disorders,^1^ characterized by persistent and significant impairments in social interaction and communication, and varying degrees of restrictive and repetitive behaviors.^1^ Evidence so far suggests that in more than 70% of individuals with ASD, there are other coexisting health, disability, or neurodevelopmental conditions present.^2,3^ In terms of ASD prevalence, estimates differ greatly across the world,^4^ due to varying techniques to identify cases, impact of sampling biases, and the cultural context.^5^ A recent study estimates that on average, around 1 in 100 children is diagnosed with ASD globally.^3^ In England, the prevalence of ASD in school children is estimated at 1.76% (95%CI 1.75%, 1.77%).^6^ While definitions of the disease, as well as instruments for detecting it have changed over the years, studies of prevalence^3,7,8^ seem to suggest it is increasing; it is unclear, however, whether this is due solely to increased recognition, shifts in definition, or partially due to increased underlying risk factors.

ASD is highly heterogeneous. Developmental trajectories are varied and span multiple behavioral dimensions, including core autistic traits and behaviors, cognition, sensitivities to sensory stimuli, social abilities, and functional skills.^5,9-11^ It is expected that young children diagnosed with ASD can potentially receive targeted interventions before developmental plasticity is lost, to foster their improved communication skills, which will provide an advantage later in life.^12^

Screening for ASD in young children has been considered.^13,14^ Because screening is an emotional experience for parents and potentially stressful for children, it is important to avoid an approach that will result in many false positives and negatives, as the consequences and emotional impact of being told a child has autism are likely to be significant.^15,16^ It is also possible that children with slow development as toddlers may improve to similar levels as their peers when older, and some evidence suggests that even children who meet ASD criteria at very young ages may improve to sub-clinical levels later on.^17,18^ Thus, screening might be inappropriate if carried out too early as it might result in false positive results due to an overtly unstable diagnosis. These considerations emphasize the importance of high sensitivity, specificity, and positive predictive values of the screening test required to justify ASD screening at young ages, as well as of the need to establish the stability of diagnosis into later childhood.

The effectiveness of early interventions is also crucial for reducing symptoms of ASD and in improving young children’s life chances. Most interventions for ASD are behavioral, and as such are costly and time-consuming. They are almost always parent-mediated, and mothers (predominantly the primary carers) are disproportionately tasked with significant roles in the process,^19^ leading to a potential loss of career and other opportunities. It is crucial that there is strong evidence to support the use of parent mediated behavioral interventions, given the intense effort involved. However, the evidence base is limited so far, and the effectiveness of early interventions has hitherto remained unclear.^13^

In 2011, the UK National Screening Committee (NSC),^13^ and in 2016 the United States Preventive Services Taskforce (USPSTF),^14^ recommended that screening should not be carried out in asymptomatic young children. Both recommendations highlighted concerns around the acceptability of screening for ASD, a lack of evidence for the benefits and harms of screening and limited evidence on the effectiveness of early interventions.^13,14^

We were commissioned to update evidence informing the UK NSC recommendation on screening for ASD in young children, specifically to address the following questions:

**Question 1:** What is the diagnostic stability of ASD in children aged under 5 years?
**Question 2:** What is the accuracy of screening tools in children under the age of 5 to identify ASD?
Are there characteristics (such as the age at which the screening test is performed) that affect accuracy?
**Question 3:** Has the benefit of early intervention in children aged 5 years and younger, detected through screening been demonstrated?

## Methods

The review protocol was registered on PROSPERO (CRD42021231868). All 3 questions were covered by a single search strategy using a combination of free-text and medical subject headings (see Additional file 1). The search was carried out on MEDLINE (via OvidSp), EMBASE (via OvidSp), CINAHL (via EBSCOhost) and PsycINFO (via OvidSp), Cochrane Database of Systematic Reviews and CENTRAL, and Clinical trials.gov. All searches were limited to articles published since 2010. Databases were searched in November 2021; an update search was conducted in August 2022. Reference lists of included studies were checked for other relevant publications.

After removing duplicates across databases, titles/abstracts, and subsequent full-texts, were reviewed against the inclusion/exclusion criteria (Table 1) by a single reviewer (either RH, BG, JW, or JP). Twenty percent of titles and abstracts were double screened. Any disagreements were resolved by discussion. Where the eligibility of an article was unclear at title and abstract screening, it was included for full-text screening to ensure that all potentially relevant studies were captured.

**Table 1.** Inclusion and Exclusion Criteria for the Key Questions.

Key question	Inclusion criteria							
	Population	Target condition	Intervention	Reference standard	Comparator	Outcome	Study type	Exclusion criteria
1	Screen detected children aged ≤ years diagnosed with ASD	ASD	NA	Any validated measure	NA	Continued diagnoses of ASD at a specified time after initial diagnosis	Longitudinal cohort studies, systematic reviews, and/or meta-analyses of these	Non-English language, published before 2010
2	Children aged ≤ 5 not diagnosed with ASD and for whom no concerns of ASD have been raised by parents, other caregivers, or clinicians	ASD	Any specific screening tool to identify ASD, performed by health visitors, GPs, parents, other non-specialist HCPs. Any general tools to identify range of conditions including ASD	Multidisciplinary team assessment and clinical judgment: NICE guidelines, SIGN guidelines. Clear reference standard as defined in the study and its standing	Any other screening tool	Sensitivity Specificity PPV NPV Incidental findings	Any test accuracy study (and systematic reviews and meta-analyses of these), with concurrent validation (reference test performed at the same time as the index test)	Non-English language, published before 2010. Case-control study design, where cases are children who already have a diagnosis of ASD
3	Children aged ≤ 5 years identified with ASD through screening, having had no previous concerns raised by parents, other caregivers, or clinicians, for ASD.				Any intervention No treatment (control group, placebo) Any intervention given after diagnostic care or routine practice (ie, not through screening)	Improvements in ASD core deficits/ symptom severity, including but not limited to: adaptive behavior, expressive language skills, receptive language skills, IQ, challenging/problem behavior, visual spatial skills, cognitive skills, academic skills, social skills, initiative behaviors	RCTs and systematic reviews of RCTs prioritized. Other study types, including cohort studies, considered if satisfactory (for example, sufficiently powered) RCTs are not available.	

Abbreviations: ASD, autism spectrum disorder; GP, general practitioner; HCP, health care professional; IQ, intelligence quotient; NICE, The National Institute for Health and Care Excellence; NPV, negative predictive value; PPV, positive predictive value.

A bespoke data extraction form was developed and piloted for each question. All data was extracted by 1 reviewer for each question, then checked by a second reviewer. Discrepancies were resolved through discussion or mediated by a third reviewer. Tools to assess the quality and risk of bias of each study included in the review were applied depending on the design of each study. We used a modification of QUIPS (the Quality in Prognostic Studies),^20^ modified versions of QUADAS-2 (Quality Assessment of Diagnostic Accuracy Studies),^21^ and QUADAS-C (for comparative accuracy studies),^22^ AMSTAR (A MeaSurement Tool to Assess systematic Reviews),^23^ the Cochrane Collaboration’s “Risk of Bias” Tool,^24^ and ROBINS-I (Risk of Bias in Non-randomized Studies—Interventions).^25^

A narrative synthesis of results is presented for each question. Where summary estimates have not been reported in studies, but raw data are available, these have been calculated.

## Results

**Figure 1.**
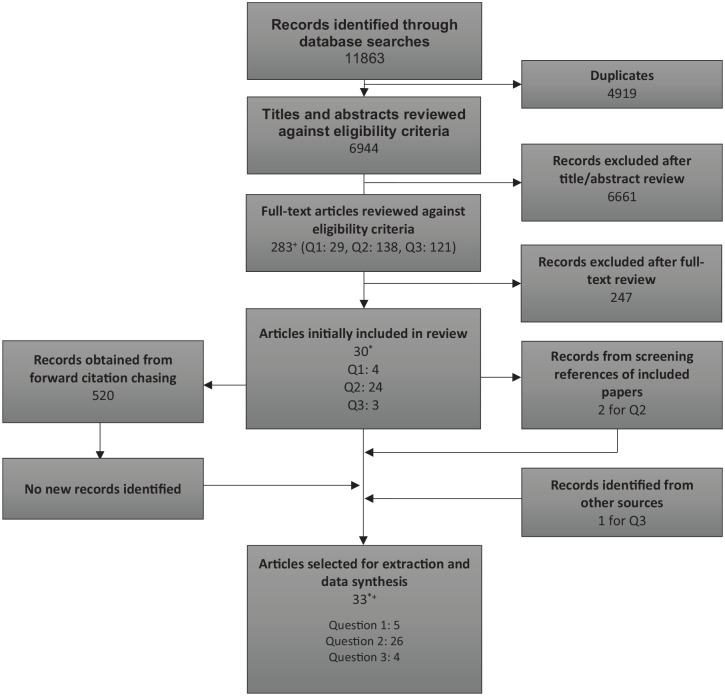
PRISMA diagram of study inclusion. *One paper included in both Q1 and Q3. ^+^One paper included in both Q1 and Q2.

Figure 1 details the number of articles identified, screened, and ultimately included in the systematic review. A total of 11 863 titles and abstracts were identified from the database searches. After de-duplication of articles from the different databases, the titles and abstracts of 6944 articles were screened using the inclusion and exclusion criteria. Two hundred and eighty-three articles were then screened at full-text, with 30 meeting our inclusion criteria. Three further articles were obtained from screening the references of those 30 articles or other sources. Thirty-three publications were ultimately judged to be relevant to 1 or more review questions (see Figure 1).

### Q1: Diagnostic Stability

Five primary studies were relevant to Q1. These were published between 2013 and 2021 and included 1580 children in total, with a mean age range between 19 and 36 months at baseline. Two studies were based in the USA,^26,27^ and 1 each in the UK,^28^ Australia,^29^ and Sweden.^30^ The time interval between diagnosis and final follow up assessment was approximately 24 months, except for the Swedish study (Spjut Jansson, 2016)^30^ which was 60 months. Follow-up assessments included less than 100 children in all studies except for Pierce,^26^ who followed up over 1200 children.

In 4 of the 5 studies, all children meeting their inclusion criteria, regardless of whether their initial diagnosis was ASD, were followed up. Spjut Jansson^30^ is the exception to this, as only children diagnosed with ASD were followed-up. A summary of study characteristics, risk of bias, and results is presented in Table 2.

**Table 2.** Characteristics, Risk of Bias, and Results for Studies Addressing Diagnostic Stability in a Population Of Children Identified by Screening.

Study	Country and population	Screening tool	N with T1 and T2 data [N with T1 only; N offered screen]	Diagnostic process at T1 and T2	Age at T1 Length of follow-up (months)	Overall risk of bias	Results (95% CI)
Allison 2021^28^	United Kingdom Registered on CHSD in Luton, Bedfordshire, and Cambridgeshire	Q-CHAT	81 [121; 13070]	T1: Experienced, psychologist(s) performed the ADOS, ADI-R, MSEL, VABS. ICD-10 criteria. T2: as above	~24 [median] NR (≥48 months old)	P: Low A: High DA T1: Low DA T2: Low C: High Blind^a^: No	100% (66.4, 100)^b^ retained possible autism diagnosis.
Pierce2019^26^	United States 75% children identified as “at-risk” from a screened population, 25% referred population	CSBS IT checklist	1269 [2241; NR]	T1: Experienced, registered psychologists performed the ADOS-2, MSEL, VABS. T2: as above	19 [mean] 20.2 [mean]	P: Low A: High DA T1: Low DA T2: Low C: High Blind^a^: No	84% (80, 87) retained ASD dx Change to ASD: 47% ASD features, 24% DD, 16% LD, 4% TD
Barbaro 2017^29^	Australia Children identified as “at-risk” from a screened population	Failing 3 of 5 behavioral items from the SACS	77 [99; >20,000]	T1: Developmental history, previous check-ups, ADOS-G Module 1, MSEL, ADI—R, FYI, CSBS IT, EDI, CHAT-23, expert clinical judgment T2: as above but excluding ADI-R	24 [time of scheduled check-up] 24	P: Unclear A: High DA T1: High DA T2: High C: Low Blind^a^: Yes	71.9% (53.2, 86.2)^b^ retained ASD dx. 40% (22.7%, 59.4%)^b^ retained autism dx. Change to ASD: 56.6% autism, 0% DD, 0% LD.
Spjut Jansson 2016^30^	Sweden Children identified as “at-risk” from routine ASD population screening	NR	71 [100; NR]	T1: Multidisciplinary assessment, including cognitive/intellectual tests, ADOS-G and DISCO (for 72% of the children). Experienced professionals. T2: As above plus ADI	Approx. 36 [mean] Approx. 60	P: High A: High DA T1: High DA T2: Low C: High Blind^a^: No	93% (84.3, 97.7)^a^ retained ASD dx.
Guthrie 2013^27^	Australia Two-step screened population	First step: CSCB IT or parental concern. Second step: CSBS red flags for ASD using SORF.	82 [unclear; 5419]	T1: ADOS-T, video-recordings, home observations, parent reports, MSEL, VABS, consistent with DSM-IV criteria by experienced clinician T2: as above	19 [mean] 16 [mean]	P: Low A: Unclear DA T1: Unclear DA T2: Unclear C: Low Blind^a^: No (all details from T1 available at T2)	100% (93.6, 100)^a^ retained ASD dx. Change to ASD: 21% deferred dx. 0% ASD ruled-out

Abbreviations: A, attrition; ADI-R, Autism Diagnostic Interview—Revised; C, confounding; CHAT-23, Checklist for Autism in Toddlers-23; CHSD Child Health Surveillance Database; CSBS, Communication and Symbolic Behavior Scales; CSBS IT, Communication and Symbolic Behavior Scales Infant-Toddler Checklist; DA, diagnostic assessment; DD, developmental delay; dx, diagnosis; EDI, Early Development Interview; FYI, First Year Inventory; LD, language delay; MSEL, Mullen Scales of Early Learning; NR, not reported; P, participants; Q-CHAT, Quantitative Checklist for Autism in Toddlers; SACS, Social Attention and Communication Study; T1, time 1; T2, time 2; TD, typically developing; SORF, Systematic Observation of Red Flags; VABS, Vineland Adaptive Behavior Scales.

a Blind, Were individuals conducting the diagnostic assessment at T2 blinded to the details and/or findings of the diagnostic assessment at T1?.

b 95% confidence intervals calculated by review authors.

In the studies providing estimates on the stability of a diagnosis of ASD over time in a screened population, these ranged from 71.9% to 100%. All studies raised concerns regarding risk of bias, including the lack of blinding of assessments at follow-up, participant attrition, clearly described methods of diagnosis, and a relatively small number of children evaluated at each time-point. In 1 study the population of screen-detected cases was mixed with clinically referred cases of ASD. Tt was unclear whether children received treatment during follow-up in some of the studies. Furthermore, 1 study which reported 100% stability,^27^ allowed for diagnosis to be deferred, suggesting that this estimate of 100% stability may not reflect the more difficult diagnoses. In fact, at follow-up (T2), 71% of those with a deferred diagnosis at baseline (T1) had been deemed as not having a diagnosis of ASD. The length of follow-up, which was 24 months for all but 1 study,^27^ are another limitation of the findings from these studies.

### Q2: Screening Accuracy Studies

Twenty-five primary studies were relevant for this question, including 1 study that was reported in 2 articles.^31,32^

The included studies evaluated the performance of 11 screening tools. These tools and relevant findings are summarized in Table 3.

**Table 3.** Summary of Screening Tools Evaluated in the Included Studies.

Screening tool in our review	Target condition(s)	Main areas covered	Intended age	Format	Time required	Source	Number of evaluation studies included
M-CHAT (-R/F)^a,b^	ASD	Early joint-attention/theory of mind, early language and communication, motor abnormalities, sensory abnormalities, and social interchange	16-30 months	2-stage: 1st parent/carer completed questionnaire 2nd parent/carer interview with health professional	5-20 min	Robins 2014^33^ Magan-Maganto2017^34^ Thabtah2019,^35^ Ozgur 2020,^36^ Sturner 2022,^37^ Zhang 2022^38^	18
Quantitative Checklist for Autism in Toddlers (Q-CHAT)^b^	ASD	Items from CHAT and additional items	18-24 months	Parent/carer-completed questionnaire (25 items)	15-20 min	Allison 2021^28^ Thabtah 2019^35^	2
Global Developmental screen (GDS)	Global development	Gross and fine motor skills, language and communication, and contains an emotional-social domain	3-60 months	Parent/carer interview with health professional, observation	NR	Kerub 2020^39^	1
Social Attention Communication Surveillance—Revised (SACS-R)	ASD	Social attention and communication	12-60 months	Observation	NR	Mozolic-Staunton2020,^40^ Barbaro 2022,^41^ Shrestha 2021^42^	3
Parents Evaluation of Developmental Status (PEDS)	Global development, ASD pathway developed	Behavior, motor skills, expressive/receptive language development, social-emotional development, and concerns around school for those children attending school	0-8 years	Parent/carer interview with health professional	5-10 min	Mozolic-Staunton 2020^40^ Thabtah ^35^	2
Social Communication Questionnaire (SCQ)	ASD	Social interaction and communication, and repetitive and stereotyped behaviors	48 months	Parent/carer questionnaire	10-20 min	Thabtah 2019^35^	1
Three-item Direct Observation Screen (TIDOS)	ASD	Joint attention, eye contact and responsiveness to name	Unclear	Observation	“no additional time” to routine check	Topcu 2018^43^	1
Ages and Stages Questionnaire (ASQ-3)	Global development	Communication, gross motor, fine motor, problem-solving, and personal-social development	0-60 months	Parent/carer questionnaire	NR	Catino 2017^44^	1
First Year Inventory (FYI)	ASD	Social-communication and sensory-regulatory domains	12 months	Parent/carer questionnaire	20-35 min	Ben-Sasson 2013,^45^ Thabtah 2019^35^	2
Infant Toddler Checklist (ITC)	Social and communication delays	Language predictors	6-24 months	Parent/carer questionnaire or interview format	5-10 min	Wieckowski 2021,^46^ Thabtah 2019^35^	1
Joint Attention Observation schedule (JA-OBS)	ASD	Joint attention	20-48 months	Observation	5-10 min	Nygren 2012^47^ Magan-Maganto 2017^34^	1
Binomial Observation Test (BOT)	NR	Responds to name and follows commands	NR	Observation	5 min	Zhang 2022^38^	1

a M-CHAT/F, original M-CHAT with follow-up interview; M-CHAT-R/F, revised M-CHAT with follow-up interview. The M-CHAT/F was first published in 2001, and a revised version, M-CHAT-R/F published in 2014^33^.

b Q-CHAT and M-CHAT(-R/F) are both based on a version of the CHAT.

All 25 studies report screening for ASD in a community based population; in many cases, screening was part of routine surveillance appointments. Six studies were based in the USA, 4 in Turkey, 2 each in Israel, Spain, and Australia, and 1 study in each of the following: the UK, Chile, China, Iceland, Japan, Italy, France, Sweden, and Nepal.

The age of screened children was between the of 12 and 36 months in most (18) of the studies. Mozolic-Staunton et al^40^ reported screening children at the ages of 12, 18, 24, and 36 to 60 months, while Catino et al^44^ screened children at 42 and 48 months old. Most of the studies were prospective cohort studies; Achenie et al,^48^ Dai et al,^49^ and Mozolic-Staunton et al^40^ reported re-analyses of previous cohort studies. Achenie et al^48^ conducted a retrospective analysis of data prospectively collected by Robins et al.^33^

The studies were generally found to be of low risk of bias on QUADAS-2 for the patient selection, index test, and reference standard domains. A number of studies were carried out with the aim of validating the M-CHAT(R/F) screening tool in populations where English is not the first language; this required the translation of the M-CHAT(R/F). Timing and patient flow were domains on which many studies were deemed to be at a high risk of bias. Design choices were generally justified by the complexity of the diagnostic evaluation for ASD, making it time and resource intensive. Consequently, children who were deemed screen-negative either did not have a diagnostic evaluation,^36,40,42,44,49-51^ only had an evaluation if they were screen-positive on another tool(s) and/or a health professional raised a concern for possible ASD diagnosis,^28,33,38,41,46-48,52-54^ had their medical records checked for a diagnosis of ASD at a later date (after 10 months,^39^ after 24 months,^31,32^ or at an unknown time-point^55^). In 4 studies, a sample of children who were deemed to be screen-negative received a diagnostic evaluation,^37,43,45,56^ while Suren et al^57^ took a number of different approaches. Although a practical constraint, only offering a diagnostic evaluation to those children who screen positive can lead to biased accuracy estimates (depending on the approach taken and the calculation and reporting of summary accuracy measures). One particular challenge of these study designs was blinding the diagnostic assessment to the screening result, as only screen-positive children received a diagnostic evaluation.

Where estimates of sensitivity were reported, those for M-CHAT(R/F) ranged from 0.67 to 1.0, with many studies reporting sensitivity estimates of around 0.8 depending on age group or cut-off used (see Table 4).

**Table 4.** Summary of Studies Evaluating the Screening Accuracy of Q-CHAT and Versions of M-CHAT.

Study, country	Intended age (months) at screening [mean age at screening, SD]	Screening tool [language] Cut-off for referral	Uptake (%, N) [ASD prevalence]	Reference standard [diagnostic criteria. tools/measures. Personnel]	Follow-up of screen negatives	Risk of bias and applicability concerns^a^	Results (95% CI)
Allison 2021^28^ UK	18-30 months	Q-CHAT [English] Decreasing probability of referral with 100% referred who ≥37 (observed) & ≥ 44 (imputed), to 1% referred with scores ≤ 37	Screening: 28.8%, 3770 Uptake: 54.3%, 121 [0.98%(0.45%, 2.16%)]	ICD-10. Consensus diagnosis as possible autism or autism spectrum (if they met the ICD-10 criteria). ADOS-G, ADI-R, MSEL, VABS. Experienced research psychologist(s) and trained research assistant.	Stratified sampling approach. Children re-screened ≥ 48 months using CAST. Those >15, and any referrals for various reasons, including autism, invited for diagnostic evaluation.	PS: Low/Low IT: Low/Low FS: Low/Low FT: High	≥39: PPV 0.17 (0.08, 0.31) Sens 1.0 (0.72, 1.0) Spec 0.95 (0.92, 0.97) NPV 1.0 (0.93, 1.0)
Jonsdottir 2020,^31^ 2021^32^ Iceland	30 [31.7, 1.72]	M-CHAT-R/F [Icelandic] >2 => FUI ≥2 => refer	Screening: 72.1%, 1586 Diagnostic evaluation: 96.2%, 25 [1.22% (0.84, 1.75)]	ICD-10. Physical and neurological examination, ADOS-2, Parent interview. Pediatrician, psychologist, and social worker.	Yes. Checked databases for any ASD diagnoses up to 2 years after screening	PS: Low/Low IT: Low/High RS: High/Low FT: High	Sens: 0.62 (0.44, 0.80) Spec: 0.99 (0.99, 1.00) PPV: 0.72 (0.51, 0.88) NPV: 0.99 (0.99, 1.00)
Magan-Maganto 2020^55^ Spain	18 and 24 [approx. 24, range 14-36]	M-CHAT-R/F [Spanish] >7 => refer 3-7 => FUI. ≥2 after FUI => refer	Screening: 56.6%, 6515 (Complete FUI 78.3%) Diagnostic evaluation: 61.3%, 19 [0.29%]	DSM-V. Clinical history, Merril-Palmer Revised Scales, Leiter, Vineland Scales, ADOS-G module 1 and ADOS-2 module T and 1. Trained and experienced professionals	Yes. Reviewed any ASD diagnoses in children who screened negative.	PS: Low/Low IT: Low/High RS: Unclear/Low FT: High	All ages Sens 0.79 (0.54,0.93) Spec 0.99 (0.99,0.99) 14-22 months Sens 0.82 (0.48-0.97) Spec 0.99 (0.99,0.99) 23-36 months Sens 0.75 (0.36-0.96) Spec 0.99 (0.99,0.99)
Oner 2020^50^ Turkey	16-36 [26.75, 5.76]	M-CHAT-R/F [Turkish] >2 =>FUI. >2 FUI => refer	Screening: 74.5%, 6712 (but denominator included those out of age range) (complete FUI 84.3%) Diagnostic evaluation: 68.8%,152 [0.8%]	DSM-V. “All available information” ADOS-2, Denver Developmental Screening-II. Study author, research certified for ADOS-2 use.	No	PS: High/Unclear IT: Low/High RS: Unclear/Unclear FT: High	M-CHAT-R Sens 1.00 (0.94, 1.0)^b^ Spec 0.91 (0.90, 0.92)^b^ PPV 0.09 (0.07, 0.11)^b^ NPV 1 (0.999, 1.0)^b^ M-CHAT-R/F (calculated by 2021 review authors) Sens 1.00 (0.97, 1.00)^b^ PPV 0.26 (0.20,0.32)^b^
Kerub 2020^39^ Israel	18-36 [21.3, 3.45]	Global Developmental Screening (GDS), M-CHAT/F [Hebrew] GDS ≥ 1 => follow-up or refer. M-CHAT/F >7 => refer 3-7 => FUI. ≥2 after FUI => refer	Screening: NR, 1591 (complete FUI NR) Diagnostic evaluation: 82.3%, 70 [0.63%]	DSM-V. Child psychiatrist/neurologist.	Yes. Reviewed medical records of those screened negative (10 months later) to identify any false negatives	PS : Unclear/Unclear IT: Unclear/High RS: Unclear/Low FT: High For comparative accuracy PS: Unclear IT: Unclear RS: Unclear FT: Unclear	M-CHAT/F Sens 0.7 (0.35, 0.93) Spec 0.98 (0.97, 0.99) PPV 0.20 (0.08, 0.37) GDS Sens 0.5 (0.19, 0.81) Spec 0.998 (0.992, 0.999) M-CHAT/F plus GDS Sens 0.7 (0.35, 0.93) Spec 0.968 (0.96, 0.97)
Dai 2020^49^ US	24 [unclear]	M-CHAT/F or M-CHAT-R/F [English, results for Spanish version not included] NR	Screening: NR, 19685 (complete FUI at 18 months 77.5%, 24 months 70.2%) Diagnostic evaluation: 70.0%,390 (18 months) 62.5%, 20 (24 months) [1.03%]	DSM-IV. Demographic information, Mullen Scales of Early Learning, Vineland Adaptive Behavior Scales, ADOS-2 Toddler Module, ADOS Module 1 and 2, CARS(2). Clinical psychologist or a developmental-behavioral pediatrician	No. Re-screened those who were screen-negative at 18 months. No screen-negatives had a diagnostic evaluation, unless they subsequently screened positive.	PS: Low/Low IT: Low/Low RS: High/Low FT:Low Authors co-owners of MCHAT LLC	NR Calculated by 2021 review authors Single screen at 18 months PPV 0.52 (0.47, 0.57) Negatives rescreened at 24 months PPV 0.50 (0.27, 0.73)
Achenie 2019^48^ [based on data from Robins 2014^33^] US	16-30 [NR]	Machine learning applied to M-CHAT-R [English] >2 =>FUI. >2 FUI => refer.	14995 (Uptake not reported, see Robins 2014) [0.77%]	DSM-IV-TR. “ADOS, CARS-2, Toddler Autism Symptom Interview, Mullen Scales of Early Learning, Vineland Adaptive Behavior Scales—II, Behavioral Assessment System for Children—2, and developmental history” Psychologist/developmental pediatrician	Random sample of screen-negatives had diagnostic evaluation [see Robins]	PS: Unclear/Unclear IT: Low/Low RS: Unclear/Low FT: High	Comparable to M-CHAT-R/F. More results available.
Topcu 2018^43^ Turkey	16-38 [NR]	TIDOS, M-CHAT/F [Turkish] M-CHAT/F: ≥2 of 7 critical items or ≥3 of 23 items were positive, so => refer TIDOS: refer if one of the 3 parameters scored ≥ 1	Screening: 40.0%, 511 Diagnostic evaluation: 91.3%, 21 [0.98%]	DSM-V NR. Child psychiatrist.	Yes. Random sample of 25 children who screened negative on M-CHAT-R/F and TIDOS. Diagnostic evaluation within 2 weeks of screen for screen-positive children and 3-9 months for screen-negative children.	PS: Low/Unclear IT: Low/ High RS: High/Low FT: High For comparative accuracy PS: Low IT: Unclear RS: High FT: Unclear	M-CHAT/F Sens 0.60 (0.15, 0.95)^b^ Spec 0.97 (0.95, 0.99)^b^ PPV 0.18 (0.04, 0.46)^b^ NPV 0.995 (0.98, 1.0)^b^ TIDOS Sens 0.80 (0.28, 0.99)^b^ Spec 0.998 (0.989, 0.999)^b^ PPV 0.80 (0.28, 0.99)^b^ NPV 0.998 (0.989, 0.999)^b^ M-CHAT/F plus TIDOS Sens 1.00 (0.48, 1.00)^b^ Spec 0.90 (0.88, 0.93)^b^ PPV 0.10 (0.03, 0.21)^b^ NPV 1.00 (0.99, 1.0)^b^
Baduel 2017^52^ France	24 [24]	M-CHAT/F [French] any 3 M-CHAT items or 2 of the 6 critical items => FUI. If still indicates ASD after FUI => refer [refs Robins 2001]	Screening: NR, 1227 (complete FUI 78.7%) Diagnostic evaluation: 100%, 20 [1.47%]	NR. 2-stage process 1st: ADOS-G, Psycho Educational Profile Revised, Vineland Adaptive Behavior Scales, but trained in use of ADOS. If reached ADOS-G threshold, referred to independent team to confirm diagnosis.	Those screen-negative at 24 months followed-up at 30 and 36 months. If then screen positive, they were referred for diagnostic assessment. As were any children who screened negative, but physicians had concerns.	PS: Unclear/Unclear IT: Low/High RS: High/Low FT: High	Sens 0.67 (0.41, 0.86) Spec 0.99 (0.98, 0.99) PPV 0.6 (0.36, 0.81)^b^ NPV 0.99 (0.99, 0.99)^b^
Kondolot 2016^56^ Turkey	18-30 [23, 3]	M-CHAT [Turkish] Refer if any 2 of 6 critical items or any 3 of 23 items were positive	Screening: Approx. 50.5%, 2021 Diagnostic evaluation: 100%, 17 [0.1%]	DSM-IV-TR. CARS Child psychiatrist.	Yes. Random sample (n=48) screened negative evaluated (6-12 months after screening)	PS: Low/Low IT: Low/High RS: High/Low FT: Low	PPV: 0.12 (0.01, 0.36) Sens: 1.00 (0.16, 1.00) Spec: 0.76 (0.64, 0.86)
Wiggins 2014^54^ US	18 and 24 [21.1, range 15.2-27.0]	M-CHAT/F PEDS [English] M-CHAT/F: any 3 of 23 items were failed or any 2 of 6 critical items were failed PEDS Path A ≥2 predictive concerns PEDS Path B 1 predictive concern noted PEDS ASD ≥3 concerns. Only children who failed M-CHAT/F were referred.	Screening: NR, 3980 Diagnostic evaluation: NR, 44 [0.75%]	NR. ADI-R, ADOS, CARS, MSEL, Vineland-II, developmental and medical history questionnaire Experienced clinicians (blind to M-CHAT/F and PEDS score)	No. (Only screen negative children for whom clinicians had raised concern were followed)	PS: Unclear/Unclear IT: Low/Low RS: Unclear/Low FT: Low	M-CHAT/F PPV 0.61 (0.45, 0.76) PEDS Path A PPV 0.55 (0.39, 0.71) PEDS Path B PPV 0.75 (0.35, 0.97) PEDS ASD PPV 0.59 (0.39, 0.76) [Very few of the PEDS positive were followed-up]
Robins 2014^33^ US	18 and 24 [20.94, 3.30]	M-CHAT-R/F [English] ≥3 items on M-CHAT-R, and either ≥3 on M-CHAT-R/F, or ≥2 on M-CHAT-R/F.	Screening: NR, 16071 (complete FUI 81.9%) Diagnostic evaluation: 63.5%, 221 [0.77%]	DSM-IV-TR. “all available information and . . . clinical judgment.” “Licensed psychologist/developmental pediatrician supervising a graduate student and research assistants.”	Random sample who screened negative completed Screening Tool for Austim in Two-Year Olds (STAT) tool. If then positive offered clinical evaluation	PS: Unclear/Unclear IT: Low/Low RS: Unclear/Low FT: High	M-CHAT-R/F ≥3 Sens 0.68 (0.58, 0.75) Spec 0.99 (0.99, 0.99) PPV 0.51 (0.43, 0.59)^b^ NPV 0.997 (0.996, 0.998)^b^ M-CHAT-R/F ≥2 Sens 0.85 (0.79, 0.92) Spec 0.99 (0.99, 0.99) PPV 0.47 (0.41, 0.54)^b^ NPV 0.999 (0.998, 0.999)^b^
Chlebowski 2013^53^ US	18 and 24 [20.4, 3.1]	M-CHAT/F [English and Spanish] screening positive on 2 of 6 critical items or on 3 of 23 items overall on both the M-CHAT and M-CHAT/F.	Screening: NR, 18989 (complete FUI 74.6%) Diagnostic evaluation: 61.5%, 171 [0.5%]	DSM-IV. ADOS, ADI-R, Mullen Scales of Early Learning, Vineland Adaptive Behavior Scales, CARS. Diagnosis made by clinical judgment “licensed clinical psychologist or developmental pediatrician and a psychology doctoral student.”	Only those who screen positive on other tools or “red-flagged” by pediatrician	PS: Unclear/Low IT: Low/Low RS: Unclear/Low FT: High	PPV 0.54 (0.46, 0.61)
Nygren 2012^47^ Sweden	30 [NR]	M-CHAT/F JA-OBS [Swedish] M-CHAT: “failure” on any 3 of the 23 items or on any 2 of the 6 critical items failed => FUI. If still “failed” => refer JA-OBS: failed ≥2 items	Screening: 80%, 3999 Diagnostic evaluation: 84.3%, 54 [1.2%]	DSM-IV and ICD-10 Vineland Adaptive Behavior Scales, Autism Diagnostic Observation Schedule, Diagnostic Interview for Social and COmmunication Disorders, Language assessments, 1-h observation of the child at preschool. “experienced neuropsychiatrists, neuropediatricians (4 in total) and neuropsychologists (2 in total) with expertise in autism.”	Only those where a concern raised	PS: Low/Low IT: Low/High RS: Unclear/Low FT: High For comparative accuracy PS: Low IT: Unclear RS: Unclear FT: Unclear	M-CHAT/F alone Sens 0.77 (0.61, 0.88) PPV 0.92 (0.78, 0.98) JA-OBS alone Sens 0.86 (0.72, 0.95) PPV 0.92 (0.80, 0.98) M-CHAT/F plus JA-OBS Sens 0.96 (0.85, 0.99) PPV 0.90 (0.77, 0.96)
Canal-Bedia 2011^51^ Spain	18 and 24 [range 18-36]	M-CHAT/F [Spanish] 3 out of 23 or 2 out of the 6 critical items => FUI. If still “failed” => dx eval	Screening: NR, 2055 Diagnostic evaluation: 9.2%, 31 [0.29%]	DSM-IV. ADOS-G, Vineland Adaptive Behavior Scales, Merril- Palmer Revised Scales of Development	No	PS: Unclear/Unclear IT: Low/High RS: Unclear/Low FT: Unclear	PPV 0.19 (0.05, 0.33)
Ozgur 2020^36^ Turkey	mean age 40.72 ±20.73 (min 12, max 150 months)	M-CHAT [Turkish] 1 of the 5 items indicating high-risk	Screening: NR, 18678 Diagnostic: 82.5%, 320 [0.5%]	DSM-V: Childhood Autism Rating Scale (CARS); Denver Developmental Screening Test—II was applied for a developmental evaluation by child and adolescent psychiatrist (CAMH)	No	PS: Unclear/Unclear IT: High/Low RS: High/Low FT: Low	PPV 0.29
Sturner 2022^37^ USA	16-20 months [18.02, 0.53]	M-CHAT-R/F Q-CHAT-10 “ M-CHAT-R/F: 3 of the 5 “key” behavioral items Q-CHAT-10: NR	Screening: NR, 11876 (complete M-CHAT FUI NR) Diagnostic: 63.8%, 639	DSM-V MSEL, ADOS-2 By experienced “autism evaluators”	A sample of gender, age and location matched children negative on either screening tool underwent diagnostic assessment	PS: High/Low IT: Low/Low RS: Low/Low FT: Low	M-CHAT-R Sens 0.73 (0.61, 0.82) Spec 0.66 (0.61, 0.71) PPV 0.28 (0.22, 0.35) NPV 0.93 (0.89, 0.96) M-CHAT-R/F Sens 0.36 (0.24, 0.49) Spec 0.89 (0.85, 0.92) PPV 0.36 (0.24, 0.49) NPV 0.89 (0.85, 0.92) Q-CHAT-10 Sens 0.34 (0.23, 0.46) Spec 0.95 (0.92, 0.97) PPV 0.54 (0.39, 0.68) NPV 0.89 (0.85, 0.92)
Zhang 2022^38^ China	18-24 months [21.1, 2.71]	M-CHAT-R/F BOT M-CHAT-R: if score 8 or more => referred. If score 3-7 => FUI, if ≥2 => referred BOT: ≥1 => referred	Screening: NR, 11190 Diagnostic: 100%, 474 [0.32% (95%CI 0.23, 0.45); 0.43% (95%CI 0.32, 0.57) after 3 years]	DSM-V ADOS-2 developmental pediatricians	Wellness checks every 3-6 months. Only referred if suspected of ASD at later point	PS: Low/Low IT: Low/Low RS: High/Low FT: Low	M-CHAT-R (≥3): PPV 0.07 M-CHAT-R/F: PPV 0.31 M-CHAT—R (≥8) PPV 0.43 BOT: PPV 0.38 BOT+M-CHAT-R (≥3) PPV: 0.42

Abbreviations: ADOS-G, Autism Diagnosis Observation Schedule-Generic; BOT, Binomial Observation Test; CARS, Childhood autism rating scale; FT, flow and timing; FUI, follow-up interview for M-CHAT(-R); IT, index test (screening tool); NPV, negative predictive value; NR, not reported.; PPV, positive predictive value; PS, participant selection; RS, reference standard (diagnostic evaluation); Sens, sensitivity; Spec, specificity

a Note that studies who only report PPV and who do not follow-up any children who have a negative screen are deemed to be of low risk of bias. However, if such a study reports sensitivity and specificity, then it is deemed to be of high risk of bias.

b Estimates calculated by review authors.

The sensitivity estimates for other tools spanned the range of possible estimates from 0.07 (0.03, 0.14) for PEDS^40^ to 1.0 (0.72, 1.0) for Q-CHAT.^28^ Where only PPVs could be estimated with confidence (ie, ASD diagnoses in screen negative children was not undertaken or assumed), these ranged from <0.10 (for the ASQ^44^ and M-CHAT-R^38^) to ≥0.80 for TIDOS,^43^ M-CHAT/F, and/or JA-OBS^47^ (see Table 5).

**Table 5.** Summary of Studies Evaluating the Screening Accuracy of Tools Other Than M-CHAT(-R/F) or in Combination With M-CHAT(-R/F).

Study, Country	Intended age (months) at screening [mean, SD]	Screening tool [language] Cut-off for referral	Uptake: %, N [ASD prevalence]	Reference standard [Dx criteria tools/measures personnel]	FU screen negatives	Risk of bias and applicability concerns	Results (95% CI)
Mozolic-Staunton 2020^40^ Australia	12, 18, 24, 36-60 [range 12 -48]	SACS-R, PEDS [English] SACS: 3 key items of concern = high risk PEDS: PATH ASD = 3 or more concerns, Path A = 2 concerns, Path B = 1 concern	Screening: NR, 13417 Diagnostic evaluation: 83.3%, 205 [1.49%]	Bayley Scales of Infant Development (BSID), Autism Diagnostic Observation Schedule, 2nd Edition (ADOS 2, Autism Diagnostic Interview-Revised (ADI-R), clinical judgment. Pediatric health professionals	No. Negative on SACS-R and PEDS not FU	PS: Unclear/Unclear IT: Low/Low RS: High/Low FT: High For comparative accuracy PS: Unclear IT: Unclear RS: Unclear FT: Unclear	SACS-R PPV 0.83 (0.78, 0.88)^a^ Sens 0.82 (0.76, 0.87)^a^ Spec 0.99 (0.99, 1.00)^a^ NPV 0.99 (0.99, 1.00)^a^ PEDS PPV 0.88 (0.71, 0.98)^a^ Sens 0.07 (0.03, 0.14)^a^ Spec 0.99 (0.99, 1.00)^a^ NPV 0.99 (0.99, 1.00)^a^
Suren 2019^57^ Norway	36 [36]	SCQ [Norwegian] ≥15 for the 39 scored items. ≥11 for the 39 scored items. ≥12 for the 33 non-verbal items.	Screening: 58%, 58520 Diagnostic evaluation: NR [0.7%]	DSM-IV-TR. ADOS, ADI-R. NR.	Random sample of age-matched controls. False negative children (those with ASD who were not screen positive) were determined by checking medical records at later time-point.	PS: Low/Low IT: Low/High RS: Unclear/High FT: High	SCQ total ≥15 Sens 0.20 (0.16,0.24) Spec 0.99 (0.99,0.99) PPV 0.09 (0.07, 0.11) NPV 0.99 (0.99, 1) SCQ total ≥11 Sens 0.42 (0.37,0.47) Spec 0.89 (0.89, 0.90) PPV 0.03 (0.02, 0.03) NPV 1 (1,1) SCQ total ≥12 Sens 0.25 (0.20,0.29) Spec 0.99 (0.99, 0.99) PPV 0.16 (0.13, 0.19) NPV 1 (0.99,1) Results also given by whether child had phrased speech or no.
Catino 2017^44^ Italy	42 and 48 [younger group 42.65, 1.82; Older group 48.08, 2.62]	ASQ-3 [Italian] scored in the clinical range in 1, or more than 1 domain	Screening: 88.7%, 514 Diagnostic evaluation: 57.5%, 40 [0.39%]	“neuropsychiatric evaluation comprehensive neuropsychiatric evaluation (cognitive, neuropsychological, and psychopathological)”	No	PS: Low/Low IT: Unclear/High RS: Low/Low FT: Unclear	For ASD PPV 0.08 (0.01, 0.25)
Ben-Sasson 2013^45^ Israel	12 [12.56]	FYI [Hebrew] 94th percentile cut-off for the social domain only, or also the 88th percentile cut-off for the sensory domain.	Screening: NR, 613 Diagnostic evaluation: NR [0.8%]	None. AOSI, MSEL. “clinician with expertise in early child Development”	Yes. 60 screen-negatives followed-up.	PS: High/Low IT: Low/High RS: Unclear/Low FT: High	Sens 0.60 (0.15, 0.95) Spec 0.753 (0.64, 0.84)
Wieckowski 2021^46^ US	Initial screen: 12, 15, 18 Re-screens: 18, 24, 36	FYI (12 months), ITC (12 & 15 months), M-CHAT-R/F (≥ 15 months) [English and Spanish] Positive on either tool (if multiple tools used).Cut-offs NR.	Screening: 12 months NR, 1504 15 months NR, 1228 18 months NR, 3053 Diagnostic evaluation from initial screen at: 12 months 36.0%, 91 15 months 29.0%, 78 18 months 20.2%, 131 [2.35%]	ICD-10. ADOS-2, TASI, or ADI-R, medical, developmental, family history. Individuals supervised by supervised by a licensed psychologist, certified school psychologist, or developmental pediatrician.	Only those for whom a concern had been raised.	PS: Low/Low IT: Unclear/High RS: Unclear/Low FT: High	**Single screen at** 12 months PPV 0.22 (0.14, 0.32)^a^ Sens 0.64 (0.48, 0.81) Spec 0.95 (0.93, 0.96) NPV 0.991 15 months PPV 0.17 (0.09, 0.27)^a^ Sens 0.72 (0.52, 0.93) Spec 0.94 (0.92, 0.95) NPV 0.995 18 months PPV 0.42 (0.34, 0.51)^a^ Sens 0.74 (0.64, 0.84) Spec 0.97 (0.97, 0.98) NPV 0.993 >1 screen from 12 months PPV 0.25 (0.17, 0.35)^a^ Sens 0.81 (0.67, 0.95) Spec 0.94 (0.93, 0.95) NPV 0.995 15 months PPV 0.19 (0.11, 0.29)^a^ Sens 0.83 (0.66, 1.00) Spec 0.94 (0.92, 0.98) NPV 0.993 18 months PPV 0.44 (0.35, 0.52)^a^ Sens 0.82 (0.74, 0.91) Spec 0.97 (0.97, 0.98) NPV 0.995
Barbaro 2022 Australia	12, 18, 24, 30, 42 months	SACS-R, SACS-PR	Screened: NR, 13511 Diagnostic assessment: 357	DSM-V MSEL, ADOS-T/ ADOS-2/ ADOS-R	No	PS: Low/Low IT: Low/Low RS: High/Low FT: High	**SACS-R (12-24 mo)** Sens 0.62 (0.57-0.66) Spec 1.00 (0.99-1.00) PPV 0.83 (0.77-0.87) **SACS-PR** Sens 0.96 (0.94-0.98) Spec 0.99 (0.98-0.99) PPV 0.78 (0.73-0.82)
Shrestha 2021 Nepal	11-30 months [19.99, 5.96]	SACS-N 3 of 5 key items => referred	Screened: 1926 Diagnostic assessment: 63.63%, 11 [0.26% including all screen positive; 1.6% including only assessed screen positive	DSM-V MSEL, ADOS-2 Psychologists	No	PS: Low/Low IT: Low/Low RS: Unclear/Low FT: Low	PPV = 0.43 (only taking into account children who attended diagnostic assessment) PPV = 0.50 (taking into account all screen positive children)

Abbreviations: ASQ-3, Ages and Stages Questionnaire, version 3; FYI, first Year Inventory; ITC, Infant Toddler Checklist; SACS-N, Social Attention Communication Surveillance-Nepal; SACS-R, Social Attention Communication Surveillance-Revised; SCQ, Social Communication Questionnaire

a 95% confidence intervals calculated by review authors.

The comparative accuracy analyses between M-CHAT/F and other screening tools suggest that tools incorporating observation of the child (TIDOS and JA-OBS) tended to perform better than parent/carer reported questionnaires such as M-CHAT/F (see Tables 4 and 5). Beside non-M-CHAT(-R/F), TIDOS and JA-OBS were the only other tools with estimates of sensitivity above 0.5: 0.8 (0.28, 0.99) for TIDOS^43^ and 0.86 (0.72, 0.95) for JA-OBS.^47^

Little evidence was found on whether age or other characteristics impact on screening accuracy.^44,55,57^

In 8 studies where screening uptake could be extracted or calculated, estimates ranged from 40% to 88.7% (see Tables 4 and 5). Where the screening tool used involved 2 stages (ie, M-CHAT(-R)/F), 6 studies reported the proportion of patients with complete screening information, which ranged from 70% to 84%. Fourteen studies reported uptake for the diagnostic evaluation of screen-positive, ranging from 57.5% to 100%. Canal-Bedia et al,^51^ reported an uptake of the diagnostic evaluation of only 9.2%. Two studies reported 100% uptake, however both studies included fewer than 20 children.

### Q3: Early Interventions

114 titles were reviewed in full text for eligibility on this question. Following full-text screening, only 3 studies were eligible: 2 USA-based RCTs^58,59^ and a prospective cohort study conducted in Sweden.^30^ A fourth study, conducted in Australia, was identified from other sources.^60^

Study characteristics, risk of bias, and results reported in the 4 included studies are summarized in Table 6.

**Table 6.** Characteristics, Risk of Bias, and Results for Studies Evaluating the Effectiveness of Early Interventions.

Study, country, design	Screening	Sample size and follow-up	Intervention and control	Overall RoB	Results^a^
Baranek 2015^58^ USA RCT	Community sample, 12 months old (N = 12 000). Screened positive on FYI, or parental concerns (N = 59/2261 responses).	24 agreed to RCT. 18 eligible. 16 randomized (11 ART, 5 REIM) ~15 months old at randomization. FU post-intervention (~22 months old), diagnostic evaluation (~32 months old)	ART, parent administered (after training). Mean of 33.5 (range 20-39) total contacts (in-home + phone/email) across a 6- to 8-month period. REIM	R: Low I: Some MD: Low M: Low RB: Low Overall: Some	ART significantly associated with improved receptive language, socialization, sensory hyporesponsiveness, and “less directive parental interactive style” during the intervention period. Little evidence of any difference at 32 month FU. ASD dx at 32 months old: 36% ART, 40% REIM, 100% not randomized.
Watson 2017^59^ USA RCT	Community sample, 12 months old (N = 61 437). Screened positive on FYI (N = 280/8709 responses).	109 declined and 74 ineligibles. 97 eligible and agreed to RCT. 87 consented to randomization (45 ART, 42 REIM) ~13.7 months old at randomization. FU post-intervention (~22 months old)	ART, parent administered (after training). Mean of 24.9 (*sd* = 5.2, range 12-32) in-home sessions and 2.4 (*sd* = 3.6, range 0-15) other contacts. REIM	R: Low I: Some MD: Low M: Low RB: Low Overall: Some	No evidence that ART associated with Improved Social-Communication, Sensory‑Regulatory, Adaptive, and Autism Symptom Outcomes. ART was associated with improvements in motor skills, but the finding could just reflect regression-to the mean. Across both groups, 41% met criteria for ASD,
Spjut Jansson 2016^30^ Sweden Prospective naturalistic cohort	2.5 year old children referred following routine screening for ASD in Gothenburg (tool NR). From 2009 to 2011, 134 <4 years referred with positive screening result.	129 consented to assessment. 100 met ASD dx criteria. 71 received interventions. Approx. 36 months old at ASD dx evaluation. FU after 2 years (approx. 60 months old).	Regular Intensive Learning program Modified intensive learning program Usual care	C: Low P: Low Class: Low I: Low MD: Low M: Low RB: Low Overall: Low	Adaptive composite scores: No evidence of increase in scores over time across total sample. No evidence of any of the interventions increased scores more than another intervention. Global functioning: Evidence that scores increased over time, but no evidence that greater increases seen with any of the interventions over another.
Whitehouse 2021^60^ Australia RCT	12 months old infants referred following community wide screening in Perth and Melbourne	104 were randomized 89 included in intention-to-treat analysis at 24 months (3 years of age)	iBASIS—Video Interaction to Promote Positive Parenting (iBASIS-VIPP) Usual care	R: Some I: Some MD: Low M: Low RB: Low Overall: Some	Combined treatment effect on reducing ASD symptom severity across time points favored the intervention (ABC, −5.53; 95%CI, −∞ to −0.28; *P* = .04).

Abbreviations: ART, adapted responsive teaching; C, confounding; FU, follow-up; FYI, First Year Inventory; I, intervention(s); MD, missing data; NA, not applicable; NR, not reported; OM, outcome measurement; P, participants; R, randomization; RB, bias in reporting outcomes; RCT, randomized controlled trial; REIM, referral to early intervention and monitoring.

a Cochrane Risk of Bias tool for RCTs, ROBINS-I for cohort study.

Two RCTs^58,59^ included children from a community population in USA, who were found to be at risk of ASD according to the First Year Inventory (FYI) tool. In Baranek et al,^58^ children who had not screened positive on FYI, but had concerns raised by their parents, were also included. In both studies, children deemed to be at-risk of ASD were randomized to either receive a parent-led intervention (Adapted Responsive Teaching, ART) lasting 24 weeks, or were referred to existing services in the local communities (referral to early intervention and monitoring, REIM). ART was described as a home-based, relationship-focussed intervention that encouraged parents “to use responsive strategies during daily routines with their children, [. . .] designed to target “pivotal” behaviors (eg, social play, joint attention, arousal and attention, engagement, adaptability, and coping).”^58^ In both studies, the intervention was provided over 6 months, and included 36 planned contacts (mainly home sessions, with additional phone calls and emails) between parents and professionals experienced in child development. Participants were identified from relatively large community populations (2261 and, respectively, 8709 screen results were available); despite this, studies included relatively low samples (16 and 87 respectively). Contacted authors confirmed that the 2 studies used different samples, independent of each other.

One study^58^ found ART was significantly associated with improved receptive language, socialization, and sensory hyporesponsiveness compared to REIM. However, the larger RCT^59^ found no evidence that ART was associated with improved outcomes compared to REIM.

The Swedish prospective cohort study^30^ included children aged 2 and a half years who were referred to the Child Neuropsychiatric Clinic following a positive screen result from routine ASD screening. Children received a wide range of interventions, varied in type and intensity. Evaluation after 2 years did not show any significant differences between the interventions on the Vineland Adaptive Behavior Scale, Second Edition (VABS-II)^61^ and the Children’s Global Assessment Scale (C-GAS).^62^

Whitehouse et al^60^ included children aged 12 months who were referred mostly due to a positive result following community wide screening. Children were randomized to either the iBASIS—Video Interaction to Promote Positive Parenting (iBASIS-VIPP) intervention or usual care. Data showed a reduction in ASD symptom severity (ABC, −5.53; 95%CI, −∞ to −0.28; *P* = .04) and reduced odds of ASD classification (odds ratio, 0.18; 95%CI, 0-0.68; *P* = .02) in the intervention group, 2 years after baseline.

The quality of the included studies was generally acceptable, with some concerns due to the lack of blinding to the intervention type for the RCTs. Spjut Jansson et al^30^ presented the least overall concern of bias, however the study was not randomized. The population included in Whitehouse et al^60^ represented a mix of screened and referred participants. When contacted for clarification, authors of the study confirmed that the majority of the included children had a positive screen result, with only a minority in 1 trial site being referred beside the screened participants.

## Discussion

### Statement of Principal Findings

Overall, the evidence on screening for ASD in young children remains weak.

Regarding diagnostic stability, although studies indicated that between 72% and 100% of children with a diagnosis of ASD retained their diagnosis 2 years later, the risk of bias within these studies, in particular the lack of blinding, means that diagnostic stability is likely to be over-estimated. Additionally, there is little evidence of the stability of ASD diagnoses beyond the age of 4 or 5 years.

Regarding the accuracy of screening, this review indicates uncertainty as to the performance and uptake of screening tools to identify children with ASD. Most studies evaluated adaptations of the M-CHAT screening tool in other languages. In these studies, estimates of sensitivity for M-CHAT(R/F) ranged from 0.67 to 1. There was some evidence that tools which included observation of the child by professionals may have better accuracy than the M-CHAT(R/F). Screening uptake was also variable, ranging from 40% to 89%. However, the main limiting factors are uncertainty on the stability of diagnoses of ASD when made at young ages, and the current lack of evidence regarding the effectiveness of interventions for children identified through screening.

In terms of the effectiveness of interventions, only 4 studies evaluated interventions in young children identified through screening for ASD, the largest of which still only included 89 children. This study reported the treatment effect (reduced ASD severity) was maintained after 2 years. However, the study sample included a small number of referred patients beside the screen-detected majority, making the generalizability of these results unclear. There was no evidence of improved outcomes in the other studies. Overall, evidence of the long-term outcomes of early intervention in young children identified through screening remains limited, as the maximum follow-up among the studies identified was just 2 years.

### Strengths and Weaknesses of the Study

Particular aspects of study design limited many of the studies included in this review. For instance, a lack of blinding limited the interpretation of most of the studies that evaluated diagnostic stability and many of the screening accuracy studies. Short follow-up periods limited the extent to which diagnoses can be said to be stable beyond 2 years after diagnosis, and interventions effective after 2 years. A particular limitation of many of the screening accuracy studies is to what extent, and how, screen-negative children were followed-up, so that reliable estimates of sensitivity and specificity estimates could be obtained.

This might entail testing a random sample of children who screened negative, or a later assessment of medical records for ASD diagnosis in children who screened negative (though such an approach also carries risks of bias).

Our research question focussed on children who were asymptomatic for ASD, therefore we are not able to comment on the ability of these screening tools in children who may have symptoms for ASD, nor offer insight on the types of symptoms, or characteristics, that might prompt screening for ASD. Other research has looked at the use of such screening tools in children at higher risk of ASD, such as the younger siblings of children diagnosed with ASD.^63^ There are a number of potential benefits from screening for ASD in young children which were not included in our systematic review, such as increasing social equity from systematic screening across populations, alleviating concern in parents following a diagnosis that would not have been identified otherwise.

### Strengths and Weaknesses in Relation to Other Studies

Both the 2011 UK NSC evidence review and 2016 USPSTF review included RCTs that evaluated interventions for children ≤5 years old diagnosed with ASD, but none of these were in children who had been identified through screening for ASD. In contrast, children included in these RCTs had “significant impairments in cognition, language, and behavior,”^14^ suggesting more severe symptoms than screen detected ones; they were also outside the intended age range of the screening tools. Although evidence did suggest that interventions could be effective in children with ASD, there remains significant uncertainty regarding the applicability of these findings in a younger population of screen-detected children.

Our finding that tools which include observation of children may perform better than parent/carer-completed checklists confirms some of the findings from the 2011 UK NSC review, where surveillance of children by trained professionals was associated with high sensitivity (0.94 for children aged 3.5 years).^13^ However, use of observational screening tools compared to parent/carer-completed screening questionnaires has considerable implications for available resources.

More recent systematic reviews of the accuracy of screening tools for ASD in children have reached similar conclusions on the variability of results and limitations in study design. For example, in their systematic review on the accuracy of screening tools for ASD in children up to 12 years of age, Levy et al^64^ highlight variability in the performance of even the same tool across settings, ages, and contexts. A review of screening tools in children <2 years old by Petrocchi et al^65^ reports similar conclusions on the variability of accuracy estimates across studies, and calls for more studies evaluating tools in the general population “with the purpose for making a diagnostic evaluation.” Although reporting that their meta-analysis results indicate accuracy of screening tools for children aged 14-36 months, Sanchez-Garcia point out that over half of the studies were deemed to be at high risk of bias, and comment on the challenge of following-up those children who have a negative screen result.

### Unanswered Questions and Future Research

To reduce uncertainty in the evidence for ASD screening in young children, better designed studies are needed. Evidence on the stability of ASD diagnoses beyond 4 or 5 years old is lacking. Further studies on diagnostic stability should consider longer follow-up periods, so that the diagnostic assessment can be made at primary school age. Ideally, follow-up assessments should be blinded to previous assessments. To evaluate accuracy of screening tools, more studies should attempt to follow-up a proportion of children who screen negative, in order to improve the reliability of sensitivity and specificity estimates. Again, measures should be taken too blind the diagnostic evaluation to the screening results. Comparative accuracy of more than 1 tool would be useful, especially when questionnaire-based tools are included together with tools involving child observation. Research is also needed on factors influencing the uptake of ASD screening and diagnostic assessment. Another relevant aspect is resource use associated with the screening tools; current evidence suggest there is a trade-off, with parent/carer questionnaires requiring relatively few resources, while approaches featuring behavioral observation by professionals needing significantly more resources, if training of such professionals is taken into account.

Designing of the ideal study to evaluate early interventions in a screened population is challenging. While large studies are usually preferred, because they provide the power to accurately evaluate intervention effects. However, the low prevalence of ASD means that, in order to achieve the required power, the population to screen would need to be very large.

Beside the low prevalence of ASD, another problem is attrition. This is particularly important in screened populations, because of many ways in which participants could drop-out. Our review found that screening uptake ranged from 40% to 88%, and could be as low as 57.5% for the subsequent diagnostic evaluation of children at risk.

These 2 issues are illustrated in the study by Watson et al:^59^ more than 8700 children were screened in order to identify a sample of 102 participants needed to detect a statistically significant difference, but only 87 consented and were available to randomization.

Thus, screening of larger or multiple populations might be necessary to ensure adequate power for evaluating the effectiveness of the interventions. Measures to mitigate attrition should also be considered. Finally, longer follow-up would allow a better evaluation of long-term outcomes following early interventions.

## Conclusions

ASD is highly heterogenous, and a wide range of cognitive, learning, language, medical, emotional and behavioral problems co-occur to variable degrees. Screening to identify ASD has been discussed for many years and, as this systematic review has shown, a number of screening tools are available. Although this update review identified new studies reporting on diagnostic stability of ASD, screening tools accuracy and early intervention effectiveness, the evidence remains unclear and insufficient to support ASD screening in young children. Better designed studies are needed to reduce uncertainty on the stability of ASD diagnoses, the accuracy of screening tools and the effectiveness of early interventions in children <5 years old for whom no concerns of ASD have been previously identified.

## Supplemental Material

sj-docx-1-jpc-10.1177_21501319241263223 – Supplemental material for Screening for Autism Spectrum Disorder in Young Children: Still Not Enough Evidence Supplemental material, sj-docx-1-jpc-10.1177_21501319241263223 for Screening for Autism Spectrum Disorder in Young Children: Still Not Enough Evidence by Bogdan Grigore, Jaime Peters, Jessica Williams, Ginny Russell, Paula Coles, Cristina Visintin, Morwenna Rogers, Robert Hayward, Zhivko Zhelev, Stuart Logan and Christopher Hyde in Journal of Primary Care & Community Health

## References

[1] American Psychiatric Association. Diagnostic and Statistical Manual of Mental Disorders. American Psychiatric Association; 2013.

[2] Lai MC Lombardo MV Baron-Cohen S. Autism. Lancet. 2014;383(9920):896-910. 24074734 10.1016/S0140-6736(13)61539-1

[3] Zeidan J Fombonne E Scorah J , et al. Global prevalence of autism: a systematic review update. Autism Res. 2022;15(5):778-790. 35238171 10.1002/aur.2696 PMC9310578

[4] Chiarotti F Venerosi A. Epidemiology of autism spectrum disorders: a review of worldwide prevalence estimates since 2014. Brain Sci. 2020;10(5):274. 32370097 10.3390/brainsci10050274 PMC7288022

[5] Elsabbagh M. Linking risk factors and outcomes in autism spectrum disorder: is there evidence for resilience? BMJ 2020;368:l6880. 31992555 10.1136/bmj.l6880

[6] Roman-Urrestarazu A van Kessel R Allison C Matthews FE Brayne C Baron-Cohen S. Association of race/ethnicity and social disadvantage with autism prevalence in 7 million school children in England. JAMA Pediatr. 2021;175(6):e210054. 10.1001/jamapediatrics.2021.0054 PMC8008434 33779707

[7] Elsabbagh M Divan G Koh YJ , et al. Global prevalence of autism and other pervasive developmental disorders. Autism Res. 2012;5(3):160-179. 22495912 10.1002/aur.239 PMC3763210

[8] Russell G Stapley S Newlove-Delgado T , et al. Time trends in autism diagnosis over 20 years: a UK population-based cohort study. J Child Psychol Psychiatry. 2022;63(6):674-682. 34414570 10.1111/jcpp.13505

[9] Doshi-Velez F Ge Y Kohane I. Comorbidity clusters in autism spectrum disorders: an electronic health record time-series analysis. Pediatrics. 2014;133(1):e54-e63. 10.1542/peds.2013-0819 PMC3876178 24323995

[bibr10-21501319241263223] Anttila V Bulik-Sullivan B Finucane HK , et al. Analysis of shared heritability in common disorders of the brain. Science. 2018;360(6395):eaap8757. 10.1126/science.aap8757 PMC6097237 29930110

[11] Jokiranta-Olkoniemi E Cheslack-Postava K Sucksdorff D , et al. Risk of psychiatric and neurodevelopmental disorders among siblings of probands with autism spectrum disorders. JAMA Psychiatry. 2016;73(6):622-629. 27145529 10.1001/jamapsychiatry.2016.0495

[12] Estes A Munson J Rogers SJ Greenson J Winter J Dawson G. Long-term outcomes of early intervention in 6-year-old children with autism spectrum disorder. J Am Acad Child Adolesc Psychiatry. 2015;54(7):580-587. 26088663 10.1016/j.jaac.2015.04.005 PMC4475272

[13] Allaby M Sharma M. Screening for Autism Spectrum Disorders on Children below the age of 5 years: a draft report for the UK National Screening Committee. UK National Screening Committee; 2011.

[14] Siu AL Bibbins-Domingo K Grossman DC , et al. Screening for autism spectrum disorder in young children: US preventive services task force recommendation statement. JAMA. 2016;315(7):691-696. 26881372 10.1001/jama.2016.0018

[15] Poslawsky IE Naber FB Van Daalen E Van Engeland H. Parental reaction to early diagnosis of their children’s autism spectrum disorder: an exploratory study. Child Psychiatry Hum Dev. 2014;45(3):294-305. 23959534 10.1007/s10578-013-0400-z

[16] Russell G Norwich B. Dilemmas, diagnosis and de-stigmatization: parental perspectives on the diagnosis of autism spectrum disorders. Clin Child Psychol Psychiatry. 2012;17(2):229-245. 22219019 10.1177/1359104510365203

[17] Turner LM Stone WL. Variability in outcome for children with an ASD diagnosis at age 2. J Child Psychol Psychiatry. 2007;48(8):793-802. 17683451 10.1111/j.1469-7610.2007.01744.x

[18] Russell G Golding J Norwich B Emond A Ford T Steer C. Social and behavioural outcomes in children diagnosed with autism spectrum disorders: a longitudinal cohort study. J Child Psychol Psychiatry. 2012;53(7):735-744. 22111544 10.1111/j.1469-7610.2011.02490.x

[19] Catalano D Holloway L Mpofu E. Mental health interventions for parent carers of children with autistic spectrum disorder: practice guidelines from a critical interpretive synthesis (CIS) systematic review. Int J Environ Res Public Health. 2018;15(2):341. 29443933 10.3390/ijerph15020341 PMC5858410

[20] Hayden JA van der Windt DA Cartwright JL Côté P Bombardier C. Assessing bias in studies of prognostic factors. Ann Intern Med. 2013;158(4):280-286. 23420236 10.7326/0003-4819-158-4-201302190-00009

[21] Whiting PF Rutjes AW Westwood ME , et al. QUADAS-2: a revised tool for the quality assessment of diagnostic accuracy studies. Ann Intern Med. 2011;155(8):529-536. 22007046 10.7326/0003-4819-155-8-201110180-00009

[22] Yang B Mallett S Takwoingi Y , et al. QUADAS-C: a tool for assessing risk of bias in comparative diagnostic accuracy studies. Ann Intern Med. 2021;174(11):1592-1599. 34698503 10.7326/M21-2234

[23] Shea BJ Reeves BC Wells G , et al. AMSTAR 2: a critical appraisal tool for systematic reviews that include randomised or non-randomised studies of healthcare interventions, or both. BMJ. 2017;358:j4008. 10.1136/bmj.j4008 PMC5833365 28935701

[24] Higgins JP Altman DG Gøtzsche PC , et al. The Cochrane Collaboration’s tool for assessing risk of bias in randomised trials. BMJ. 2011;343:d5928. 10.1136/bmj.d5928 PMC3196245 22008217

[25] Sterne JA Hernán MA Reeves BC , et al. ROBINS-I: a tool for assessing risk of bias in non-randomised studies of interventions. BMJ. 2016;355:i4919. 10.1136/bmj.i4919 PMC5062054 27733354

[26] Pierce K Gazestani VH Bacon E , et al. Evaluation of the diagnostic stability of the early autism spectrum disorder phenotype in the general population starting at 12 months. JAMA Pediatr. 2019;173(6):578-587. 31034004 10.1001/jamapediatrics.2019.0624 PMC6547081

[27] Guthrie W Swineford LB Nottke C Wetherby AM. Early diagnosis of autism spectrum disorder: stability and change in clinical diagnosis and symptom presentation. J Child Psychol Psychiatry. 2013;54(5):582-590. 23078094 10.1111/jcpp.12008 PMC3556369

[28] Allison C Soufer R Baron-Cohen S , et al. Quantitative checklist for autism in toddlers (Q-CHAT). A population screening study with follow-up: the case for multiple time-point screening for autism. BMJ Paediatr Open. 2021;5(1):e000700. 10.1136/bmjpo-2020-000700 PMC8166626 34131593

[29] Barbaro J Dissanayake C. Diagnostic stability of autism spectrum disorder in toddlers prospectively identified in a community-based setting: behavioural characteristics and predictors of change over time. Autism. 2017;21(7):830-840. 27474118 10.1177/1362361316654084

[30] Spjut Jansson B Miniscalco C Westerlund J Kantzer A-K Fernell E Gillberg C . Children who screen positive for autism at 2.5 years and receive early intervention: a prospective naturalistic 2-year outcome study. Neuropsychiatr Dis Treat. 2016;12:2255-2263. 27621636 10.2147/NDT.S108899 PMC5012621

[31] Jonsdottir SL Saemundsen E Gudmundsdottir S Haraldsdottir GS Palsdottir AH Rafnsson V. Implementing an early detection program for autism in primary healthcare: screening, education of healthcare professionals, referrals for diagnostic evaluation, and early intervention. Res Autism Spect Disord. 2020;77:101616.

[32] Jonsdottir SL Saemundsen E Jonsson BG Rafnsson V. Validation of the modified checklist for autism in toddlers, revised with follow-up in a population sample of 30-month-old children in iceland: a prospective approach. J Autism Dev Disord. 2022;52(4):1507-1522. 33945117 10.1007/s10803-021-05053-1

[33] Robins DL Casagrande K Barton M Chen CM Dumont-Mathieu T Fein D. Validation of the modified checklist for Autism in toddlers, revised with follow-up (M-CHAT-R/F). Pediatrics. 2014;133(1):37-45. 24366990 10.1542/peds.2013-1813 PMC3876182

[34] Magán-Maganto M Bejarano-Martín Á Fernández-Alvarez C , et al. Early Detection and intervention of ASD: a European overview. Brain Sci. 2017;7(12):159. 29194420 10.3390/brainsci7120159 PMC5742762

[35] Thabtah F Peebles D. Early autism screening: a comprehensive review. Int J Environ Res Public Health. 2019;16(18):3502. 31546906 10.3390/ijerph16183502 PMC6765988

[36] Ozgur BG Aytac H. Outcomes of autism spectrum disorder screening and follow-up program in a sample of Turkey. Psychiatry Clin Psychopharmacol. 2020;30(3):241-247.

[37] Sturner R Howard B Bergmann P , et al. Autism screening at 18 months of age: a comparison of the Q-CHAT-10 and M-CHAT screeners. Mol Autism. 2022;13(1):2. 34980240 10.1186/s13229-021-00480-4 PMC8722322

[38] Zhang Y Zhou Z Xu Q , et al. Screening for autism spectrum disorder in toddlers during the 18-and 24-month well-child visits. Front Psychiatry. 2022;13:879625. 35573353 10.3389/fpsyt.2022.879625 PMC9097214

[39] Kerub O Haas EJ Meiri G Davidovitch N Menashe I. A comparison between two screening approaches for ASD among toddlers in Israel. J Autism Dev Disord. 2020;50(5):1553-1560. 30099656 10.1007/s10803-018-3711-x

[40] Mozolic-Staunton B Donelly M Yoxall J Barbaro J. Early detection for better outcomes: universal developmental surveillance for autism across health and early childhood education settings. Res Autism Spect Disord. 2020;71:101496.

[41] Barbaro J Sadka N Gilbert M , et al. Diagnostic accuracy of the social attention and communication surveillance-revised with preschool tool for early autism detection in very young children. JAMA Netw Open. 2022;5(3):e2146415. 10.1001/jamanetworkopen.2021.46415 PMC8917423 35275169

[42] Shrestha R Dissanayake C Barbaro J. Implementing and evaluating Social Attention and Communication Surveillance (SACS) to prospectively identify autism in very young children in Nepal. Res Dev Disabil. 2021;115:104013. 34144316 10.1016/j.ridd.2021.104013

[43] Topcu S Ulukol B Oner O Simsek Orhon F Baskan S. Comparison of tidos with m-chat for screening autism spectrum disorder. Psychiatry Clin Psychopharmacol. 2018;28(4):416-422.

[44] Catino E Di Trani M Giovannone F , et al. Screening for developmental disorders in 3- and 4-year-old italian children: a preliminary study. Front Pediatr. 2017;5:181. 28900613 10.3389/fped.2017.00181 PMC5581879

[45] Ben-Sasson A Carter AS. The application of the first year inventory for ASD screening in Israel. J Autism Dev Disord. 2012;42(9):1906-1916. 22234796 10.1007/s10803-011-1436-1

[46] Wieckowski AT Hamner T Nanovic S , et al. Early and repeated screening detects autism spectrum disorder. J Pediatr. 2021;234:227-235. 33711288 10.1016/j.jpeds.2021.03.009 PMC8238814

[bibr47-21501319241263223] Nygren G Sandberg E Gillstedt F Ekeroth G Arvidsson T Gillberg C. A new screening programme for autism in a general population of Swedish toddlers. Res Dev Disabil. 2012;33(4):1200-1210. 22502846 10.1016/j.ridd.2012.02.018

[48] Achenie LEK Scarpa A Factor RS Wang T Robins DL McCrickard DS . A machine learning strategy for autism screening in toddlers. J Dev Behav Pediatri. 2019;40(5):369-376. 10.1097/DBP.0000000000000668 PMC6579619 30985384

[49] Dai YG Miller LE Ramsey RK Robins DL Fein DA Dumont-Mathieu T. Incremental utility of 24-month autism spectrum disorder screening after negative 18-month screening. J Autism Dev Disord. 2020;50(6):2030-2040. 30830489 10.1007/s10803-019-03959-5 PMC6722033

[bibr50-21501319241263223] Oner O Munir KM. Modified checklist for autism in toddlers revised (MCHAT-R/F) in an urban metropolitan sample of young children in Turkey. J Autism Dev Disord. 2020;50(9):3312-3319. 31414260 10.1007/s10803-019-04160-4 PMC7065507

[51] Canal-Bedia R Garcia-Primo P Martin-Cilleros MV , et al. Modified checklist for autism in toddlers: cross-cultural adaptation and validation in Spain. J Autism Dev Disord. 2011;41(10):1342-1351. 21161677 10.1007/s10803-010-1163-z

[52] Baduel S Guillon Q Afzali MH Foudon N Kruck J Roge B. The French version of the modified-checklist for autism in toddlers (M-CHAT): a validation study on a French sample of 24 month-old children. J Autism Dev Disord. 2017;47(2):297-304. 27817161 10.1007/s10803-016-2950-y

[bibr53-21501319241263223] Chlebowski C Robins DL Barton ML Fein D. Large-scale use of the modified checklist for autism in low-risk toddlers. Pediatrics. 2013;131(4):e1121-e1127. 10.1542/peds.2012-1525 PMC3608483 23530174

[54] Wiggins LD Piazza V Robins DL. Comparison of a broad-based screen versus disorder-specific screen in detecting young children with an autism spectrum disorder. Autism. 2014;18(2):76-84. 23262658 10.1177/1362361312466962 PMC4384166

[55] Magan-Maganto M Canal-Bedia R Hernandez-Fabian A , et al. Spanish cultural validation of the modified checklist for autism in toddlers, revised. J Autism Dev Disord. 2020;50(7):2412-2423. 30328577 10.1007/s10803-018-3777-5

[56] Kondolot M Ozmert EN Oztop DB Mazicioglu MM Gumus H Elmali F. The modified checklist for autism in Turkish toddlers: a different cultural adaptation sample. Res Autism Spect Disord. 2016;21:121-127.

[57] Suren P Saasen-Havdahl A Bresnahan M , et al. Sensitivity and specificity of early screening for autism. BJPsych Open. 2019;5(3):e41. 10.1192/bjo.2019.34 PMC6537444 31530312

[58] Baranek GT Watson LR Turner-Brown L , et al. Preliminary efficacy of adapted responsive teaching for infants at risk of autism spectrum disorder in a community sample. Autism Res Treat. 2015;2015:386951. 25648749 10.1155/2015/386951 PMC4306223

[59] Watson LR Crais ER Baranek GT , et al. Parent-mediated intervention for one-year-olds screened as at-risk for autism spectrum disorder: a randomized controlled trial. J Autism Dev Disord. 2017;47(11):3520-3540. 28861651 10.1007/s10803-017-3268-0

[60] Whitehouse AJO Varcin KJ Pillar S , et al. Effect of preemptive intervention on developmental outcomes among infants showing early signs of autism: a randomized clinical trial of outcomes to diagnosis. JAMA Pediatr. 2021;175:e213298. 10.1001/jamapediatrics.2021.3298 PMC8453361 34542577

[61] Sparrow SS Cichetti DV Balla DA. Vineland Adaptive Behaviour Scales. 2nd ed. American Guidance Service; 2005.

[62] Schorre BE Vandvik IH. Global assessment of psychosocial functioning in child and adolescent psychiatry. a review of three unidimensional scales (CGAS, GAF, GAPD). Eur Child Adolesc Psychiatry. 2004;13(5):273-286. 15490275 10.1007/s00787-004-0390-2

[63] Bradbury K Robins DL Barton M , et al. Screening for autism spectrum disorder in high-risk younger siblings. J Dev Behav Pediatr. 2020;41(8):596-604. 32576788 10.1097/DBP.0000000000000827 PMC7572497

[64] Levy SE Wolfe A Coury D , et al. Screening tools for autism spectrum disorder in primary care: a systematic evidence review. Pediatrics. 2020;145(Supplement_1):S47-S59. 10.1542/peds.2019-1895H 32238531

[65] Petrocchi S Levante A Lecciso F. Systematic review of level 1 and level 2 screening tools for autism spectrum disorders in toddlers. Brain Sci. 2020;10(3):180. 32204563 10.3390/brainsci10030180 PMC7139816

