# Supplementary material for: Screening for Autism Spectrum Disorder in Young Children: Still Not Enough Evidence
Source: J Prim Care Community Health. 2024 Jul 30;15:21501319241263223. doi: 10.1177/21501319241263223 (PMC11289826; doi:10.1177/21501319241263223)
Supplement: sj-docx-1-jpc-10.1177_21501319241263223 – Supplemental material for Screening for Autism Spectrum Disorder in Young Children: Still Not Enough Evidence [file sj-docx-1-jpc-10.1177_21501319241263223.docx]

**Appendix**

Search strategies

MEDLINE

Database: Ovid MEDLINE(R) ALL <1946 to November 13, 2020>

Search Strategy:

--------------------------------------------------------------------------------

1 exp Autism Spectrum Disorder/di, rh, th [Diagnosis, Rehabilitation, Therapy] (10860)

2 autis*.ti,jn. (33767)

3 ASD.ti. (1736)

4 asperger*.ti. (1126)

5 Developmental Disabilities/di [Diagnosis] (5139)

6 Neurodevelopmental Disorders/di [Diagnosis] (439)

7 Child Development Disorders, Pervasive/di [Diagnosis] (2110)

8 ((developmental or neurodevelopment*) adj (condition* or disorder*)).ti,ab. (19779)

9 or/1-4 (37836)

10 or/1-8 (58015)

11 Child, Preschool/ (925828)

12 (child* or preschool* or toddler* or infant*).ti,ab. (1695387)

13 ("young child*" or toddler* or infant* or preschool*).ti,ab. (477666)

14 11 or 12 (2112390)

15 11 or 13 (1297535)

16 test*.ti,ab. (3264770)

17 M CHAT.ti,ab. (144)

18 PDDST.ti,ab. (3)

19 autism spectrum quotient.ti,ab. (479)

20 scale.ti,ab. (744294)

21 checklist.ti,ab. (36389)

22 tool*.ti,ab. (751429)

23 index.ti,ab. (797646)

24 questionnaire*.ti,ab. (525966)

25 screening.ti,ab. (536834)

26 instrument*.ti,ab. (288019)

27 (measure or measures or measuring).ti,ab. (1450192)

28 observation schedule.ti,ab. (694)

29 or/16-28 (6521202)

30 "sensitivity and specificity"/ or "predictive value of tests"/ (521015)

31 stability.ti,ab. (436796)

32 diagnostic value.ti,ab. (34170)

33 sensitivity.ti,ab. (815702)

34 specificity.ti,ab. (471234)

35 (validity or validation).ti,ab. (364353)

36 reliability.ti,ab. (168571)

37 (utility or utilisation or utilization).ti,ab. (413254)

38 predictive value.ti,ab. (93819)

39 accuracy.ti,ab. (414727)

40 acceptability.ti,ab. (37814)

41 feasibility.ti,ab. (188628)

42 false positives.ti,ab. (13433)

43 false negatives.ti,ab. (6254)

44 or/30-43 (2988350)

45 (early adj3 intervention*).ti,ab. (34422)

46 play therapy.ti,ab. (392)

47 attention intervention*.ti,ab. (58)

48 communication intervention*.ti,ab. (872)

49 language intervention*.ti,ab. (469)

50 (play adj2 intervention*).ti,ab. (585)

51 pivotal response.ti,ab. (79)

52 occupational therap*.ti,ab. (13889)

53 applied behavio?r analysis.ti,ab. (550)

54 focus?ed behavio?ral intervention*.ti,ab. (28)

55 psychosocial intervention*.ti,ab. (5464)

56 or/45-55 (56129)

57 10 and 14 and 29 and 44 (3311)

58 9 and 15 and 56 (885)

59 57 or 58 (4076)

60 limit 59 to yr="2010-Current" (2980)

EMBASE

Database: Embase <1974 to 2020 November 13>

Search Strategy:

--------------------------------------------------------------------------------

1 exp autism/di [Diagnosis] (8154)

2 autis*.ti,jn. (41834)

3 ASD.ti. (3042)

4 asperger.ti. (873)

5 developmental disorder/di [Diagnosis] (3716)

6 1 or 2 or 3 or 4 (47146)

7 1 or 2 or 3 or 4 or 5 (50331)

8 preschool child/ (554471)

9 (child* or preschool* or toddler* or infant*).ti,ab. (2082239)

10 (young child* or toddler* or infant* or preschool*).ti,ab. (551188)

11 8 or 9 (2300984)

12 8 or 10 (1022028)

13 exp autism assessment/ (2387)

14 test*.ti,ab. (4435690)

15 M CHAT.ti,ab. (223)

16 pddst.ti,ab. (5)

17 autism spectrum quotient.ti,ab. (600)

18 scale.ti,ab. (1006270)

19 checklist.ti,ab. (49785)

20 tool*.ti,ab. (1018185)

21 questionnaire*.ti,ab. (771359)

22 screening.ti,ab. (757364)

23 instrument*.ti,ab. (374360)

24 (measure or measures or measuring).ti,ab. (1912691)

25 observation schedule.ti,ab. (921)

26 or/13-25 (8083317)

27 "sensitivity and specificity"/ (375166)

28 diagnostic value/ (191675)

29 predictive value/ (180074)

30 stability.ti,ab. (502558)

31 diagnostic value.ti,ab. (45177)

32 sensitivity.ti,ab. (1063611)

33 specificity.ti,ab. (611490)

34 (validity or validation).ti,ab. (495009)

35 reliability.ti,ab. (209048)

36 (utility or utilisation or utilization).ti,ab. (565201)

37 predictive value.ti,ab. (143699)

38 accuracy.ti,ab. (537700)

39 acceptability.ti,ab. (49072)

40 feasibility.ti,ab. (267784)

41 false positives.ti,ab. (18496)

42 false negatives.ti,ab. (8725)

43 or/27-42 (3729059)

44 (early adj3 intervention*).ti,ab. (52087)

45 play therapy.ti,ab. (506)

46 attention intervention*.ti,ab. (73)

47 communication intervention*.ti,ab. (1143)

48 language intervention*.ti,ab. (564)

49 (play adj2 intervention*).ti,ab. (818)

50 pivotal response.ti,ab. (97)

51 occupational therap*.ti,ab. (20451)

52 applied behavio?r analysis.ti,ab. (504)

53 focus?ed behavio?ral intervention*.ti,ab. (40)

54 psychosocial intervention*.ti,ab. (7911)

55 or/44-54 (83080)

56 7 and 11 and 26 and 43 (3034)

57 6 and 12 and 55 (967)

58 56 or 57 (3870)

59 limit 58 to yr="2010-Current" (3015)

Database: APA PsycInfo <1806 to November Week 2 2020>

Search Strategy:

--------------------------------------------------------------------------------

1 autism spectrum disorders/ (44700)

2 autis*.ti,jn. (36049)

3 ASD.ti. (1586)

4 asperger*.ti. (1955)

5 developmental disabilities/ (12483)

6 ((developmental or neurodevelopment*) adj (condition* or disorder* or disabilit*)).ti,ab. (21312)

7 1 or 2 or 3 or 4 (46609)

8 1 or 2 or 3 or 4 or 5 or 6 (65981)

9 exp preschool students/ (11882)

10 (child* or preschool* or toddler* or infant*).ti,ab. (748517)

11 ("young child*" or toddler* or infant* or preschool*).ti,ab. (153233)

12 9 or 10 (748763)

13 9 or 11 (154849)

14 M CHAT.ti,ab. (140)

15 PDDST.ti,ab. (6)

16 autism spectrum quotient.ti,ab. (432)

17 scale.ti,ab. (318583)

18 checklist.ti,ab. (26568)

19 tool*.ti,ab. (155799)

20 index.ti,ab. (102522)

21 questionnaire*.ti,ab. (280262)

22 screening.ti,ab. (65318)

23 instrument*.ti,ab. (139778)

24 (measure or measures or measuring).ti,ab. (553395)

25 observation schedule.ti,ab. (749)

26 or/14-25 (1199756)

27 test reliability/ (54772)

28 test performance/ (4840)

29 test validity/ (79303)

30 stability.ti,ab. (45321)

31 diagnostic value.ti,ab. (1433)

32 sensitivity.ti,ab. (93311)

33 specificity.ti,ab. (37213)

34 (validity or validation).ti,ab. (169924)

35 reliability.ti,ab. (86436)

36 (utility or utilisation or utilization).ti,ab. (92000)

37 predictive value.ti,ab. (7850)

38 accuracy.ti,ab. (74361)

39 acceptability.ti,ab. (13869)

40 feasibility.ti,ab. (22897)

41 false positives.ti,ab. (1628)

42 false negatives.ti,ab. (670)

43 or/27-42 (533772)

44 (early adj3 intervention*).ti,ab. (19381)

45 play therapy.ti,ab. (3056)

46 attention intervention*.ti,ab. (68)

47 communication intervention*.ti,ab. (784)

48 language intervention*.ti,ab. (910)

49 (play adj2 intervention*).ti,ab. (762)

50 pivotal response.ti,ab. (183)

51 occupational therap*.ti,ab. (10345)

52 applied behavio?r analysis.ti,ab. (1869)

53 focus?ed behavio?ral intervention*.ti,ab. (14)

54 psychosocial intervention*.ti,ab. (5497)

55 or/44-54 (41727)

56 8 and 12 and 26 and 43 (2920)

57 7 and 13 and 55 (1020)

58 56 or 57 (3817)

59 limit 58 to yr="2010 -Current" (2673)

**CINAHL**

| **#** | **Query** | **Results** |
| --- | --- | --- |
| S57 | S55 OR S56 | 1,111 |
| S56 | S8 AND S13 AND S54 | 369 |
| S55 | S9 AND S14 AND S28 AND S43 | 1,086 |
| S54 | S44 OR S45 OR S46 OR S47 OR S48 OR S49 OR S50 OR S51 OR S52 OR S53 | 39,673 |
| S53 | TI "psychosocial intervention*" OR AB "psychosocial intervention*" | 3,296 |
| S52 | TI ( "focused behavior" or "focused behaviour" or "focussed behavior" or "focussed behaviour" ) OR AB ( "focused behavior" or "focused behaviour" or "focussed behavior" or "focussed behaviour" ) | 25 |
| S51 | TI ( "applied behavior analysis" or "applied behaviour analysis" ) OR AB ( "applied behavior analysis" or "applied behaviour analysis" ) | 298 |
| S50 | TI "occupational therap*" OR AB "occupational therap*" | 24,349 |
| S49 | TI "pivotal response" OR AB "pivotal response" | 69 |
| S48 | TI play N2 intervention OR AB play N2 intervention | 519 |
| S47 | TI "early intervention*" OR AB "early intervention*" | 10,404 |
| S46 | TI "attention intervention*" OR AB "attention intervention*" | 41 |
| S45 | TI "communication intervention*" OR AB "communication intervention*" | 727 |
| S44 | TI "play therapy" OR AB "play therapy" | 422 |
| S43 | S29 OR S30 OR S31 OR S32 OR S33 OR S34 OR S35 OR S36 OR S37 OR S38 OR S39 OR S40 OR S41 OR S42 | 474,710 |
| S42 | TI "false negatives" OR AB "false negatives" | 910 |
| S41 | TI "false positives" OR AB "false positives" | 1,885 |
| S40 | TI "feasibility" OR AB "feasibility" | 47,818 |
| S39 | TI "acceptability" OR AB "acceptability" | 15,529 |
| S38 | TI "accuracy" OR AB "accuracy" | 75,004 |
| S37 | TI "predictive value" OR AB "predictive value" | 22,794 |
| S36 | TI ( "utility" or "utilisation" or "utilization" ) OR AB ( "utility" or "utilisation" or "utilization" ) | 101,858 |
| S35 | TI "reliability" OR AB "reliability" | 61,558 |
| S34 | TI ( "validity" or "validation" ) OR AB ( "validity" or "validation" ) | 108,295 |
| S33 | TI "specificity" OR AB "specificity" | 57,182 |
| S32 | TI "sensitivity" OR AB "sensitivity" | 113,584 |
| S31 | TI "diagnostic value" OR AB "diagnostic value" | 4,923 |
| S30 | TI "stability" OR AB "stability" | 37,139 |
| S29 | (MM "Sensitivity and Specificity") | 1,894 |
| S28 | S15 OR S16 OR S17 OR S18 OR S19 OR S20 OR S21 OR S22 OR S23 OR S24 OR S25 OR S26 OR S27 | 1,047,705 |
| S27 | TI "observation schedule" OR AB "observation schedule" | 326 |
| S26 | TI ( "measure" or "measures" or "measuring" ) OR AB ( "measure" or "measures" or "measuring" ) | 421,034 |
| S25 | TI "instrument" OR AB "instrument" | 44,204 |
| S24 | TI "screening" OR AB "screening" | 134,809 |
| S23 | TI "questionnaire" OR AB "questionnaire" | 171,214 |
| S22 | TI "index" OR AB "index" | 203,292 |
| S21 | TI "tool*" OR AB "tool*" | 178,549 |
| S20 | TI "checklist" OR AB "checklist" | 17,142 |
| S19 | TI "scale" OR AB "scale" | 204,357 |
| S18 | TI "autism spectrum quotient" OR AB "autism spectrum quotient" | 178 |
| S17 | TI "PDDST" OR AB "PDDST" | 2 |
| S16 | TI "M Chat" OR AB "M Chat" | 83 |
| S15 | (MM "Diagnosis, Developmental") | 254 |
| S14 | S10 OR S11 | 572,626 |
| S13 | S10 OR S12 | 139,222 |
| S12 | TI ( "young child*" or preschool* or toddler* or infant* ) OR AB ( "young child*" or preschool* or toddler* or infant* ) | 139,085 |
| S11 | TI ( child* or preschool* or toddler* or infant* ) OR AB ( child* or preschool* or toddler* or infant* ) | 572,562 |
| S10 | (MM "Child, Preschool") | 385 |
| S9 | S1 OR S2 OR S3 OR S4 OR S5 OR S6 OR S7 | 25,862 |
| S8 | S1 OR S2 OR S3 OR S4 | 23,675 |
| S7 | TI "developmental disorder*" OR AB "developmental disorder*" | 2,207 |
| S6 | (MM "Child Development Disorders, Pervasive/DI") | 323 |
| S5 | (MM "Developmental Disabilities/DI") | 881 |
| S4 | TI asperger* | 982 |
| S3 | TI ASD | 1,418 |
| S2 | TI autis* OR SO autis* | 22,192 |
| S1 | (MM "Autistic Disorder/DI") | 2,496 |

Cochrane Library

Search Name: Autism screening

Date Run: 17/11/2020 16:35:01

Comment:

ID Search Hits

#1 MeSH descriptor: [Autistic Disorder] explode all trees 1001

#2 autis*:ti,ab 3484

#3 ASD:ti 321

#4 asperger*:ti 40

#5 MeSH descriptor: [Developmental Disabilities] explode all trees 635

#6 MeSH descriptor: [Neurodevelopmental Disorders] explode all trees and with qualifier(s): [diagnosis - DI] 1026

#7 MeSH descriptor: [Child Development Disorders, Pervasive] explode all trees and with qualifier(s): [diagnosis - DI] 176

#8 ((developmental or neurodevelopment*) NEXT (condition* or disorder*)):ti,ab 784

#9 #1 or #2 or #3 or #4 3624

#10 #1 or #2 or #3 or #4 or #5 or #6 or #7 or #8 5330

#11 MeSH descriptor: [Child, Preschool] explode all trees 29316

#12 (child* or preschool* or toddler* or infant*):ti,ab 153535

#13 ("young child*" or toddler* or infant* or preschool*):ti,ab 47657

#14 #11 or #12 159396

#15 #11 or #13 71371

#16 test*:ti,ab 332015

#17 M CHAT:ti,ab 175

#18 PDDST:ti,ab 0

#19 "autism spectrum quotient":ti,ab 34

#20 scale:ti,ab 157276

#21 checklist:ti,ab 6494

#22 tool*:ti,ab 31669

#23 index:ti,ab 132731

#24 questionnaire*:ti,ab 106632

#25 screening:ti,ab 51010

#26 instrument*:ti,ab 21709

#27 (measure or measures or measuring):ti,ab 203711

#28 "observation schedule":ti,ab 114

#29 #16 or #17 or #18 or #19 or #20 or #21 or #22 or #23 or #24 or #25 or #26 or #27 or #28 685023

#30 MeSH descriptor: [Sensitivity and Specificity] explode all trees 15343

#31 MeSH descriptor: [Predictive Value of Tests] explode all trees 6960

#32 stability:ti,ab 14138

#33 "diagnostic value":ti,ab 801

#34 sensitivity:ti,ab 45526

#35 specificity:ti,ab 10947

#36 (validity or validation):ti,ab 18779

#37 reliability:ti,ab 8781

#38 (utility or utilisation or utilization):ti,ab 27621

#39 "Predictive value":ti,ab 5441

#40 accuracy:ti,ab 17227

#41 acceptability:ti,ab 14637

#42 feasibility:ti,ab 34185

#43 "false positives":ti,ab 461

#44 "false negatives":ti,ab 250

#45 #30 or #31 or #32 or #33 or #34 or #35 or #36 or #37 or #38 or #39 or #40 or #41 or #42 or #43 or #44 167707

#46 early NEXT/3 intervention*:ti,ab 4818

#47 "play therapy":ti,ab 98

#48 "attention intervention*":ti,ab 30

#49 "communication intervention*":ti,ab 258

#50 "language intervention*":ti,ab 108

#51 play NEXT/2 intervention:ti,ab 126

#52 "pivotal response":ti,ab 44

#53 "occupational therapies":ti,ab 13

#54 "applied behaviour analysis":ti,ab or "applied behavior analysis":ti,ab 65

#55 ("focused behaviour intervention*" or "focused behavior intervention*" or "focussed behaviour intervention*" or "focussed behavior intervention*"):ti,ab 0

#56 "psychosocial intervention*":ti,ab 1118

#57 #46 or #47 or #48 or #49 or #50 or #51 or #52 or #53 or #54 or #55 or #56 6539

#58 #10 and #14 and #29 and #45 441

#59 #9 and #15 and #57 223

#60 #58 or #59 638

Clinical Trials

screening | autism | Child

**Appraisal for quality and risk of bias**

Quality assessments of included studies are reported below.

Table 7. Quality assessment of studies relevant to Question 1 (after QUIPS)

| Reference | Participants | | | Attrition | | | | | | Diagnosis Assessment at T1 | | |
| --- | --- | --- | --- | --- | --- | --- | --- | --- | --- | --- | --- | --- |
|  | **Representative sample** | **Avoided inappropriate exclusions** | **Overall RoB** | **Adequate response rate** | **Details of drop-outs** | **Reasons for loss-to-follow-up** | **Described lost participants** | **No important differences** | **Overall RoB** | **Clear definition/**  **description** | **Method and setting same** | **Overall RoB** |
| Screened population | |  |  |  |  |  |  |  |  |  |  |  |
| Allison 2021(28) | Yes | Yes | Low | No | Yes | Yes | Yes | Unclear | No | Yes | Yes | Low |
| Pierce 2019(26) | Yes | Yes | Low | Yes | No | Yes | Unclear | Unclear | High | Yes | Yes | Low |
| Barbaro 2017(29) | Yes | Unclear | Unclear | No | Unclear | No | Unclear | Unclear | High | No | Yes | High |
| Spjut Jansson 2016(30) | Yes | No | High | No | No | Yes | No | Unclear | High | No | No | High |
| Guthrie 2013(27) | Yes | Yes | Low | Yes | NA | NA | NA | NA | Low | Yes | Unclear | Unclear |
| Non-screened population | |  |  |  |  |  |  |  |  |  |  |  |
| McDonald 2020(66) | No | Unclear | High | No | NR | Yes | Yes | Unclear | High | No | Yes | High |
| Pellicano 2012(67) | No | Unclear | High | No | ? | Yes | Unclear | Yes | High | Yes | Yes | Low |
| Soke 2011(68) | No | No | High | No | No | No | Unclear | Unclear | High | Yes | Yes | Low |
| Anglim 2020(69) | No | No | High | No | Unclear | No | No | Unclear | High | No | No | High |

RoB, risk of bias

Table 8. Quality assessment of studies relevant to Question 1 (after QUIPS) continued

| Reference | Diagnostic assessment at T2 | | | Confounding | | |  |  |
| --- | --- | --- | --- | --- | --- | --- | --- | --- |
|  | **Clear definition/description** | **Method and setting same** | **Overall RoB** | **Measured important confounders** | **Clear definitions** | **Overall RoB** | **Blinding** | **Pre-specified design** |
| Screened population | |  |  |  |  |  |  |  |
| Allison 2021(28) | Yes | No | High | No | No | High | Yes | Yes |
| Pierce 2019(26) | Yes | Yes | Low | No | No | High | No | Unclear |
| Barbaro 2017(29) | No | Yes | High | Yes | Yes | Low | Yes | Yes |
| Spjut Jansson 2016(30) | Yes | Yes | Low | No | No | High | No | Unclear |
| Guthrie 2013(27) | Yes | Unclear | Unclear | Yes | Yes | Low | No | Yes |
| Non-screened population | |  |  |  |  |  |  |  |
| McDonald 2020(66) | Yes | No | High | No | No | High | Yes | Yes |
| Pellicano 2012(67) | Yes | No | High | Yes | No | High | Unclear | Yes |
| Soke 2011(68) | Yes | Yes | Low | No | No | High | No | Yes |
| Anglim 2020(69) | No | No | High | No | No | High | Unclear | Yes |

RoB, risk of bias

Table 9. Quality assessment of screening accuracy studies relevant to Question 2

|  | Consecutive or random sample? | Avoided a case-control design? | Avoid inappropriate exclusions? | Risk of bias for selection of patients? | Concerns of applicability of included patients? | Index test results blind to reference standard? | Pre-specified threshold? | Risk of bias for index test? | Concerns of applicability of index test? | Reference standard correctly classify target condition? | Reference standard results blind to index test? | Risk of bias for reference standard? | Concerns of applicability of reference standard? | Appropriate interval between index test(s) and reference standard? | All patients receive a reference standard? | Did patients receive the same reference standard? | Were all patients included in the analysis? | Risk of bias for patient flow? | Was the study method pre-specified? | Is the study funded/conducted by screening tool developers? |
| --- | --- | --- | --- | --- | --- | --- | --- | --- | --- | --- | --- | --- | --- | --- | --- | --- | --- | --- | --- | --- |
| Allison 2021(28) | Y | Y | Y | Low | Low | Y | Y | Low | Low | Y | Y | Low | Low | U | N | Y | Y | High | Y | N |
| Jonsdottir 2021, 2020(31, 32) | Y | Y | Y | Low | Low | Y | Y | Low | High | Y | N | High | Low | N | N | Y | N | High | U | N |
| Wieckowski 2021(46) | Y | Y | Y | Low | Low | U | U | U | High | Y | U | U | Low | U | N | Y | Y | High | Y | N |
| Kerub 2020(49) | Y | Y | U | U | U | Y | U | U | High | Y | U | U | Low | U | N | U | N | High | U | N |
| Magan-Maganto 2020(50) | Y | Y | Y | Low | Low | Y | Y | Low | High | Y | U | U | Low | U | N | U | N | High | U | N |
| Oner 2020(38) | N | Y | U | High | U | Y | Y | Low | High | U | U | U | U | U | N | Y | N | High | U | N |
| Mozolic-Staunton 2020(33) | U | Y | U | U | U | Y | Y | Low | Low | Y | N | High | Low | Y | N | U | N | High | U | N |
| Dai 2020(36) | Y | Y | Y | Low | Low | Y | Y | Low | Low | Y | N | High | Low | Y | N | Y | Y | Low* | U | Y |
| Achenie 2019(35) | Y | Y | U | U | U | Y | Y | Low | Low | Y | U | Unclear | Low | U | N | Y | N | High | U | N |
| Suren 2019(55) | Y | Y | Y | Low | Low | Y | Y | Low | High | N | U | U | High | U | N | N | Y | High | U | N |
| Topcu 2018(52) | Y | Y | Y | Low | U | Y | Y | Low | High | Y | N | High | Low | Y | N | Y | N | High | U | N |
| Catino 2017(34) | Y | Y | Y | Low | Low | Y | N | U | High | Y | U | Low | Low | U | N | Y | U | U* | U | N |
| Baduel 2017(42) | Y | Y | U | U | U | Y | Y | Low | High | Y | N | High | Low | U | N | Y | N | High | U | N |
| Kondolot 2016(53) | Y | Y | Y | Low | Low | Y | Y | Low | High | Y | N | High | Low | Y | N | Y | Y | Low | U | N |
| Wiggins 2014(47) | Y | Y | U | U | U | Y | Y | Low | Low | Y | U | U | Low | U | N | Y | Y | Low* | U | N |
| Robins 2014(37) | Y | Y | U | U | U | Y | Y | Low | Low | Y | U | U | Low | U | N | Y | N | High | U | N |
| Ben-Sasson 2013(51) | Y | Y | N | High | Low | Y | Y | Low | High | U | N | U | Low | U | N | Y | N | High | U | N |
| Chlebowski 2013(43) | Y | Y | N | U | Low | Y | Y | Low | Low | Y | U | U | Low | U | N | Y | N | High | U | N |
| Nygren 2012(44) | Y | Y | Y | Low | Low | Y | Y | Low | High | Y | U | U | Low | U | N | Y | N | High | U | N |
| Canal-Bedia 2011(39) | Y | Y | U | U | U | Y | Y | Low | High | Y | U | U | Low | U | N | Y | U | U | U | N |
| Barbaro 2022 | Y | Y | Y | Low | Low | Y | Y | Low | Low | Y | N | High | Low | Y | N | N | N | High | U | N |
| Ozgur 2020 | Y | Y | U | U | Low | Y | Y | High | Low | Y | N | High | Low | U | N | Y | N | Low | U | N |
| Shrestha 2021 | Y | Y | Y | Low | Low | Y | Y | Low | Low | Y | N | High | Low | U | N | Y | Y | Low | U | N? |
| Sturner 2022 | Y | Y | N | High | Low | Y | Y | Low | Low | Y | Y | Low | Low | Y | N | Y | N | High | U | N |
| Zhang 2022 (48) | Y | Y | Y | Low | Low | Y | Y | Low | Low | Y | U | U | Low | Y | N | Y | Y | Low | U | N |

N, no; U, unclear; Y, yes; *Although not all participants received the reference standard these studies were deemed to be at low risk of bias as only PPVs were reported

Table 10. Quality assessment of the comparative screening accuracy aspect of studies relevant to Question 2

|  | Low risk of bias for selection of participants for all index tests? | Receive all index tests or to be randomly allocated to index tests? | If randomized, was the allocation sequence random? | If randomized, was the allocation sequence concealed? | Risk of bias for selection of patients? | Low risk of bias for index tests? | If patients received multiple index tests, were test results interpreted without knowledge of the results of the other index test(s)? | If patients received multiple index tests, is undergoing one index test unlikely to affect the performance of the other index test(s)? | Were differences in the conduct or interpretation between the index tests unlikely to advantage one of the tests? | Could the conduct or interpretation of the index tests have introduced bias in the comparison? | Was risk of bias for this domain judged ‘low’ for all index tests? | Did the reference standard avoid incorporating any of the index tests? | Could the reference standard, its conduct, or its interpretation have introduced bias in the comparison? | Was risk of bias for this domain judged ‘low’ for all index tests? | Was there an appropriate interval between the index tests? | Was the same reference standard used for all index tests? | Are the proportions and reasons for missing data similar across index tests? | Could the patient flow have introduced bias in the comparison? |
| --- | --- | --- | --- | --- | --- | --- | --- | --- | --- | --- | --- | --- | --- | --- | --- | --- | --- | --- |
| Kerub 2020(49) | N | Y | NA | NA | U | N | N | U | U | U | N | U | U | N | U | Y | NA | U |
| Topcu 2018(52) | Y | Y | NA | NA | Low | Y | U | U | U | U | N | Y | High | N | Y | Y | Y | U |
| Nygren 2012(44) | Y | Y | NA | NA | Low | Y | N | U | U | U | Y | Y | U | N | U | Y | NA | U |
| Sturner | Y | Y | NA | NA | Low | Y | U | U | N | U | Y | Y | U | Y | Y | Y | U | U |

N, no; U, unclear; Y, yes

Table 11. Quality assessment of randomised controlled trials relevant to Question 3 (Cochrane RoB)

|  | Baranek 2015(58) | Watson 2017(59) | Whitehouse 2021(60) |
| --- | --- | --- | --- |
| 1.1 Was the allocation sequence random? | Y (Randomization was conducted using a random number generator in Excel by an investigator blind to the assessment results) | Y (a randomization sequence was generated using a randomization method for small samples that mixes simple randomization with permuted block randomization) | Y (was performed by minimization stratified by site, sex, number of relevant behaviours, and age range at recruitment, with randomization determined by a biased coin with a probability of 0.7) |
| 1.2 Was the allocation sequence concealed until participants were enrolled and assigned to interventions? | Y (families were notified of their assignment following randomization) | Y (team members who interacted with participants were not privy to randomization method details) | Y |
| 1.3 Did baseline differences between intervention groups suggest a problem with the randomization process? | N | N | Y (beside screened population the sample from one site also included referred infants) |
| D1. Risk-of-bias judgement | Low concern | Low concern | Some concern |
| 2.1. Were participants aware of their assigned intervention during the trial? | Y (families were notified of their assignment following randomization) | Y | Y (families could not be blinded to group allocation) |
| 2.2. Were carers and people delivering the interventions aware of participants' assigned intervention during the trial? | Y | Y (Of necessity, intervention team staff then learned if a family was allocated to the ART group) | Y |
| 2.3. If Y/PY/NI to 2.1 or 2.2: Were there deviations from the intended intervention that arose because of the trial context? | NI | NI | NI |
| 2.4 If Y/PY to 2.3: Were these deviations likely to have affected the outcome? | NA | NA | NA |
| 2.5. If Y/PY/NI to 2.4: Were these deviations from intended intervention balanced between groups? | NA | NA | NA |
| 2.6 Was an appropriate analysis used to estimate the effect of assignment to intervention? | Y | Y | Y |
| 2.7 If N/PN/NI to 2.6: Was there potential for a substantial impact (on the result) of the failure to analyse participants in the group to which they were randomized? | NA | NA | NA |
| D2. Risk-of-bias judgement | Some concerns | Some concerns | Some concerns |
| 3.1 Were data for this outcome available for all, or nearly all, participants randomized? | Y | Y | Y |
| 3.2 If N/PN/NI to 3.1: Is there evidence that the result was not biased by missing outcome data? | NA | NA | NA |
| 3.3 If N/PN to 3.2: Could missingness in the outcome depend on its true value? | NA | NA | NA |
| 3.4 If Y/PY/NI to 3.3: Is it likely that missingness in the outcome depended on its true value? | NA | NA | NA |
| D3. Risk-of-bias judgement | Low concern | Low concern | Low concern |
| 4.1 Was the method of measuring the outcome inappropriate? | PN | PN | PN |
| 4.2 Could measurement or ascertainment of the outcome have differed between intervention groups? | PN | PN | PN |
| 4.3 If N/PN/NI to 4.1 and 4.2: Were outcome assessors aware of the intervention received by study participants? | N (The assessment team was blinded to group assignment; parents were instructed to not share information regarding EI services or group assignment) | N (assessment team staff remained blind to allocation throughout the project) | N (research staff conducting the assessments were independent of the clinical teams administering the iBASIS-VIPP intervention) |
| 4.4 If Y/PY/NI to 4.3: Could assessment of the outcome have been influenced by knowledge of intervention received? | NA | NA | NA |
| 4.5 If Y/PY/NI to 4.4: Is it likely that assessment of the outcome was influenced by knowledge of intervention received? | NA | NA | NA |
| D4. Risk-of-bias judgement | Low concern | Low concern | Low concern |
| 5.1 Were the data that produced this result analysed in accordance with a pre-specified analysis plan that was finalized before unblinded outcome data were available for analysis? | PY | PY | PY |
| 5.2. Is the numerical result being assessed likely to have been selected, on the basis of the results, from multiple eligible outcome measurements (e.g. scales, definitions, time points) within the outcome domain? | PN | PN | PN |
| 5.3 Is the numerical result being assessed likely to have been selected, on the basis of the results, from multiple eligible analyses of the data? | PN | PN | PN |
| D5. Risk-of-bias judgement | Low concern | Low concern | Low concern |

PN, partial no, PY, partial yes; N, no; NA, not applicable; U, unclear; Y, yes

Table 12. Quality assessment of non-randomised controlled trials relevant to Question 3 (ROBINS-I)

| Reference | Spjut Jansson 2016(30) |
| --- | --- |
| 1.1 Is there potential for confounding of the effect of intervention in this study? | N |
| 1.2. Was the analysis based on splitting participants’ follow up time according to intervention received? | NA |
| 1.3. Were intervention discontinuations or switches likely to be related to factors that are prognostic for the outcome? | NA |
| 1.4. Did the authors use an appropriate analysis method that controlled for all the important confounding domains? | Y (adaptive composite score and C-GAS before vs after treatment were used as dependent variables in 2 separate mixed analysis of variance (ANOVA) and intellectual level as independent variables in the intervention groups) |
| 1.5. [If Y/PY to 1.4]: Were confounding domains that were controlled for measured validly and reliably by the variables available in this study? | Y |
| 1.6. Did the authors control for any post-intervention variables that could have been affected by the intervention? | N |
| 1.7. Did the authors use an appropriate analysis method that controlled for all the important confounding domains and for time-varying confounding? | Y |
| Bias due to confounding judgement | Low |
| 2.1. Was selection of participants into the study (or into the analysis) based on participant characteristics observed after the start of intervention? | N (all eligible children were referred) |
| 2.2. [If Y/PY to 2.1]: Were the post-intervention variables that influenced selection likely to be associated with intervention? | NA |
| 2.3 [If Y/PY to 2.2]: Were the post-intervention variables that influenced selection likely to be influenced by the outcome or a cause of the outcome? | NA |
| 2.4. Do start of follow-up and start of intervention coincide for most participants? | Y |
| 2.5. [If Y/PY to 2.2 and 2.3, or N/PN to 2.4]: Were adjustment techniques used that are likely to correct for the presence of selection biases? | NA |
| Bias in selection of participants into the study judgement | Low |
| 3.1 Were intervention groups clearly defined? | Y |
| 3.2 Was the information used to define intervention groups recorded at the start of the intervention? | Y |
| 3.3 Could classification of intervention status have been affected by knowledge of the outcome or risk of the outcome? | PN |
| Bias in classification of interventions judgement | Low |
| 4.1. Were there deviations from the intended intervention beyond what would be expected in usual practice? | PN |
| 4.2. [If Y/PY to 4.1]: Were these deviations from intended intervention unbalanced between groups and likely to have affected the outcome? | NA |
| 4.3. Were important co-interventions balanced across intervention groups? | PY |
| 4.4. Was the intervention implemented successfully for most participants? | Y |
| 4.5. Did study participants adhere to the assigned intervention regimen? | PY |
| 4.6. [If N/PN to 4.3, 4.4 or 4.5]: Was an appropriate analysis used to estimate the effect of starting and adhering to the intervention? | NA |
| Bias due to deviations from intended interventions judgement | Low |
| 5.1 Were outcome data available for all, or nearly all, participants? | Y |
| 5.2 Were participants excluded due to missing data on intervention status? | N |
| 5.3 Were participants excluded due to missing data on other variables needed for the analysis? | N |
| 5.4 [If PN/N to 5.1, or Y/PY to 5.2 or 5.3]: Are the proportion of participants and reasons for missing data similar across interventions? | NA |
| 5.5 [If PN/N to 5.1, or Y/PY to 5.2 or 5.3]: Is there evidence that results were robust to the presence of missing data? | NA |
| Bias due to missing data judgement | Low |
| 6.1 Could the outcome measure have been influenced by knowledge of the intervention received? | PN |
| 6.2 Were outcome assessors aware of the intervention received by study participants? | N (All the professionals were blinded to the type of intervention received by the children.) |
| 6.3 Were the methods of outcome assessment comparable across intervention groups? | Y |
| 6.4 Were any systematic errors in measurement of the outcome related to intervention received? | PN |
| Bias in measurement of outcomes judgement | Low |
| 7.1.Is the reported effect estimate likely to be selected, on the basis of the results, from multiple outcome measurements within the outcome domain? | N |
| 7.2. Is the reported effect estimate likely to be selected, on the basis of the results, from multiple analyses of the intervention-outcome relationship? | N |
| 7.3. Is the reported effect estimate likely to be selected, on the basis of the results, from different subgroups? | N |
| Bias in selection of the reported result judgement | Low |

PN, partial no; PY, partial yes; N, no; NA, not applicable; Y, yes
